# Ferritinophagy and α-Synuclein: Pharmacological Targeting of Autophagy to Restore Iron Regulation in Parkinson’s Disease

**DOI:** 10.3390/ijms23042378

**Published:** 2022-02-21

**Authors:** Matthew K. Boag, Angus Roberts, Vladimir N. Uversky, Linlin Ma, Des R. Richardson, Dean L. Pountney

**Affiliations:** 1 School of Pharmacy and Medical Science, Griffith University, Gold Coast 4222, Australia; m.boag@griffith.edu.au (M.K.B.); angus.roberts@griffithuni.edu.au (A.R.); 2 Griffith Institute of Drug Discovery, Griffith University, Nathan 4111, Australia; linlin.ma@griffith.edu.au (L.M.); d.richardson@griffith.edu.au (D.R.R.); 3 Morsani College of Medicine, University of South Florida, Tampa, FL 33612, USA; vuversky@usf.edu; 4 Centre for Cancer Cell Biology and Drug Discovery, School of Environment and Sciences, Griffith University, Nathan 4111, Australia

**Keywords:** α-synuclein, ferritin, iron, autophagy, ferritinophagy, vesicular trafficking, Parkinson’s disease, neurodegeneration

## Abstract

A major hallmark of Parkinson’s disease (PD) is the fatal destruction of dopaminergic neurons within the *substantia nigra pars compacta*. This event is preceded by the formation of Lewy bodies, which are cytoplasmic inclusions composed of α-synuclein protein aggregates. A triad contribution of α-synuclein aggregation, iron accumulation, and mitochondrial dysfunction plague nigral neurons, yet the events underlying iron accumulation are poorly understood. Elevated intracellular iron concentrations up-regulate ferritin expression, an iron storage protein that provides cytoprotection against redox stress. The lysosomal degradation pathway, autophagy, can release iron from ferritin stores to facilitate its trafficking in a process termed ferritinophagy. Aggregated α-synuclein inhibits SNARE protein complexes and destabilizes microtubules to halt vesicular trafficking systems, including that of autophagy effectively. The scope of this review is to describe the physiological and pathological relationship between iron regulation and α-synuclein, providing a detailed understanding of iron metabolism within nigral neurons. The underlying mechanisms of autophagy and ferritinophagy are explored in the context of PD, identifying potential therapeutic targets for future investigation.

## 1. Introduction

Parkinson’s disease (PD) is the most prevalent degenerative motor disorder, characterized by dysfunction and death of dopaminergic neurons within the *substantia nigra pars compacta* (SNpc) [1,2,3,4,5]. The intrinsically disordered synaptic protein, α-synuclein, is the principal component of neuronal Lewy bodies (LB) and Lewy neurites (LN), which are cytoplasmic inclusions that hallmark α-synucleinopathies. Protein aggregation, mitochondrial dysfunction, and intracellular iron accumulation converge in a triad pathology [3,6,7,8] that progressively disperses across the neocortex [9,10,11,12].

Both iron and α-synuclein promote mitochondrial dysfunction, yet the aetiology of iron deregulation remains poorly understood [13]. Upon increased iron concentration within cells, up to 4500 Fe^3+^ ions per unit can be stored within ferritin, a universal intracellular iron storage protein [14,15,16,17,18]. Ferritinophagy is a subtype of the autophagy-lysosomal pathway and is the only known mechanism by which iron bound to ferritin can be released [19,20,21]. However, this degradative pathway depends on functional vesicular trafficking and membrane fusion events, which become inhibited by α-synuclein aggregates. Strong evidence suggests that dysfunctional ferritinophagy can potentiate iron overload in nigral neurons [21]. This review provides a comprehensive description of the role of α-synuclein in vesicular trafficking with a focus on neuronal iron metabolism and autophagy. Current data will be outlined linking α-synuclein aggregation to the dysfunction of ferritinophagy and identifying potential targets for novel therapeutic strategies.

## 2. Roles of Iron, Calcium and α-Synuclein in Nigral Neurons

The SNpc is the most densely populated dopaminergic region, comprising 200,000 to 420,000 dopaminergic neurons, much of which die in PD patient tissue [8,22,23,24], ultimately manifesting into the cardinal symptoms of ataxia, bradykinesia, resting tremor, and a shuffling gait. Non-motor symptoms, such as cognitive dysfunction and decline, often occur at later disease stages but may also precede motor signs. In addition, both motor and cognitive dysfunction may be related to SNpc degeneration, as the cortical region is a crucial indirect regulator of voluntary motor control and behavioural learning [25].

Sensory stimuli transduction originates from the pedunculopontine nucleus (PPN), which transverses through the reticular activating system and then into the SNpc [26,27]. From here, a direct pathway exists (via dopamine D1 receptor stimulation) to propagate an excitatory stimulus for the overlying GABAergic (i.e., depending on γ-aminobutyric acid (GABA) *substantia nigra pars reticulata* (SNpr) [28]. GABAergic neurotransmission suppresses the internal globus pallidus, freeing the basolateral nuclei to stimulate the motor cortex. A parallel indirect pathway (via dopamine D2 receptors) excites GABAergic projections from the dorsal striatum. Subsequent repression of the external globus pallidus means the excitatory glutaminergic (i.e., glutamic acid neurotransmission) subthalamic nuclei can stimulate the internal globus pallidus. GABAergic signalling directed towards the thalamic basolateral nuclei completes an inhibitory circuit that regulates voluntary motor control, comprising the cortico-basal ganglia-thalamocortical loop [26]. This transduction circuit is implicated in numerous diseases, including PD, Huntington’s disease (HD), and neuropsychiatric disorders, such as attention-deficit hyperactivity disorder [29,30,31].

The SNpc is internally compartmentalized by calbindin-D28K (CALB1) expression, with an immuno-positive dorsal and negative ventral area [24,32]. In addition to selective expression of CALB1, the SNpc exhibits slow, broad oscillations that persistently supply the striatum with dopaminergic neurotransmission. Autonomous conduction stems from sporadic depolarization events that alter voltage-gated ion channels, dynamically fluctuating membrane potentials [33,34]. CALB1 probably combines with other physiological neuroprotectants (e.g., neuromelanin and metallothionein) to shield against the influx of detrimental ions. Neuromelanin (5,6-dihydroxyindole) is the darkly pigmented substance that has historically identified the SNpc [35] and is a chelator of metals and binds other reactive species [23,36]. PD histology exhibits the complete loss of SNpc pigmentation, indicating the death of all nigral neuromelanin-positive neurons [35].

## 3. Synaptic Role of α-Synuclein

**Figure 1:**
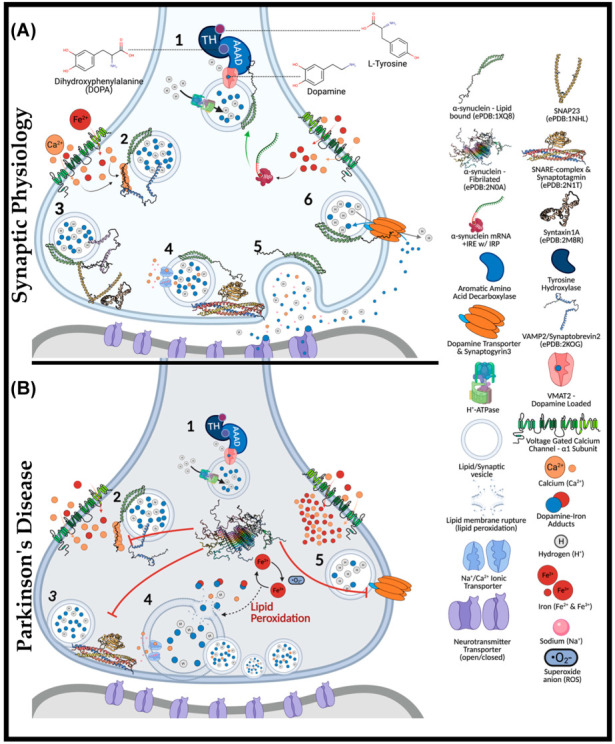
The Role of α-Synuclein at the Synaptic Junction in Physiology and in Parkinson’s Disease. In normal physiology (**A**): (1) complexed tyrosine hydroxylase (TH) and aromatic amino acid decarboxylase (AAAD) bind to the vesicular monoamine transporter (VMAT2), yielding vesicular dopamine. Vesicle membrane-bound H^+^-ATPases lower the intra-vesicular pH. Lipid-vesicle embedded α-synuclein has a role (2) in chaperoning soluble *N*-ethylmaleimide-sensitive factor attachment protein receptors (SNARE) after calcium activation. *C*-terminal binding between calcium-activated α-synuclein and vesicular-associated membrane protein 2 (VAMP2), exposes the SNARE motif for synaptosome-associated protein of 25 kDa (SNAP25) binding. SNAP25 recruits syntaxin-1A, and with the assistance of accessory proteins (synaptotagmin and Munc-18/13, an acronym for mammalian uncoordinated-18 or -13), the SNARE complex is formed (4), prompting the exocytosis of vesicular cargo (5). The α-synuclein is critical for synaptic plasticity by recycling synaptic vesicles post-exocytosis. Afterwards, (6) α-synuclein associates with the dopamine transporter to bring vesicles into proximity for synaptogyrin3 to modulate direct dopamine influx from the synaptic cleft. (**B**) (1) complexed tyrosine hydroxylase (TH) and aromatic amino acid decarboxylase (AAAD) bind to the vesicular monoamine transporter (VMAT2), yielding vesicular dopamine. Iron has also been suggested to permeate voltage-gated calcium channels (VGCCs), increasing the intracellular iron pool that may drive α-synuclein synthesis via a controversial and atypical 5′ iron-responsive element (IRE) in the untranslated region of α-synuclein mRNA. However, in the absence of lipid membrane vesicles, excessive iron-induced α-synuclein synthesis can yield disordered species that readily fibrillate (2) upon calcium exposure. The α-synuclein aggregates bind VAMP2 *C*-termini to block SNARE complex assembly (3). Consequently, vesicles cluster at the presynaptic membrane (4), while iron redox chemistry can lead to lipid peroxidation. Integral membrane damage can rupture vesicles, expelling reactive catecholamines, which lead to further damage through redox-active dopamine-iron adducts. Dopamine recycling is halted (5) as α-synuclein aggregates block vesicles docking at the dopamine transporter (DAT).

Synaptic physiology and plasticity are modulated by α-synuclein (Figure 1). Upon correct folding of α-synuclein, the lipophilic *N*-terminus folds into twin helices that embed into small phospholipid vesicles (SMV) [37,38] and is supported by the *N*-acetylmethionine modification at residue M1 [39,40]. Despite being an intrinsically disordered cytosolic protein, the *N*-terminus of α-synuclein is highly conserved, with imperfect KTKEGV repeats that promote helix formation in the lipid-bound conformation [41]. The first helix extends from residues 1–25, while the parallel strand is between residues 31–55. Aspartate (D2) and glutamate (E13 and E20) are charged amino acids that stabilize the outer-surface residues, enhancing the lipid-binding of phosphoglycerol heads at lysine residues, K6, K10, and K12, at membrane surfaces [42]. Familial mutations within the *SNCA* gene (*PARK1*), including A30P, E46K, H50Q, G51D, A53E, and A53T, with sporadic PD-associated mutants, A18T and A29S, are also being linked to PD [43,44,45,46,47,48]. These α-synuclein mutants have diminished lipid binding, propagating disequilibrium towards non-membrane-bound monomers [43,44,45,46,47,48].

Physiologically, α-synuclein is a calcium-sensing chaperone of soluble *N*-ethylmaleimide-sensitive factor attachment protein receptor (SNARE) protein complexes [49]. Calcium-binding enhances the hydrophobicity of α-synuclein for interactions with the vesicular-SNARE protein (v-SNARE or R-SNARE motif), including the vesicle-associated membrane protein 2 (VAMP2, also known as synaptobrevin) [50,51]. Synaptic exocytosis relies upon VAMP2, as demonstrated through knockout mice lacking evoked synaptic potential, thus being embryonically lethal [52,53]. VAMP2 mutations in the SNARE motif (A67P, S75P, F77S, & E77A) manifest severe neurodevelopmental disorders with cognitive deficits [54]. Interactions between calcium-bound α-synuclein (*C*-terminus) and VAMP2 (*N*-terminus) induce a structural reorganization of the latter, exposing the internal SNARE motif of VAMP2. If not already docked at presynaptic membranes, these events may parallel the Hsc70-CSPα-SGT chaperone complex [53,55,56].

Heat shock cognate 70 kDa (Hsc70) is an ATPase that covalently conjugates cysteine string protein-α (CSPα) to vesicular membranes for synaptic trafficking [56]. CSPα is another SNARE complex co-chaperone that also recruits small glutamate-rich tetratricopeptide repeat-containing protein (SGT). CSPα mechanistically serves to prime the target-SNARE (t-SNARE or Qb-SNARE-motif) and synaptosomal-associated protein of 25 kDa (SNAP25) at presynaptic membranes [57]. Interestingly, CSPα-knockout in mice causes significant neurodegeneration [58] that is rescued upon overexpressing human α-synuclein [57]. This result indicates that α-synuclein may analogously act to prime and stabilize SNAP25 in a compensatory manner. Another noteworthy point is that Hsc70 can directly bind fibrillated α-synuclein to facilitate chaperone-mediated autophagy (CMA) [59].

Unlike other SNARE proteins, SNAP25 does not contain a transmembrane domain and instead attaches to presynaptic membranes by palmitoylation at central cysteine residues [60]. The *C*- and *N*-termini of SNAP25 each contain a separate SNARE motif linking the proximal syntaxin-1a (Qa-SNARE motif) and vesicle-bound VAMP2 [61]. In attempts to prevent sporadic synaptic firing, syntaxin-1 remains independently regulated by mammalian uncoordinated 18 (Munc-18) and Munc-13 [62]. Munc-18 halts exocytosis by altering the conformation of syntaxin-1a. The Habc domain of synatxin-1a is rearranged to block exogenous interactions with the protein’s SNARE motif. Upon stimulation, Munc-13 switches with Munc-18 through their MUN domain (residues 29–96), ultimately releasing the folded syntaxin-1 for SNAP25 interactions [61,63]. It is unclear whether VAMP2 or α-synuclein may facilitate Munc-18/13 interchange. At this point, α-synuclein remains free of any known binding partners, whereas VAMP2 is complexed. Thus, it is more likely α-synuclein participates in the switching of Munc-13/18. To complete neurotransmitter release, the SNARE motifs of VAMP2, syntaxin-1, and SNAP25 (*trans*-SNARE-complex) yield a half-zipper conformation that is structurally aided by the synaptotagmin protein family and complexins (*SNAREpins)*.

Each component of the SNARE complexes is individually regulated (i.e., syntaxin-1 with Munc-13-Munc-18, SNAP25 with CSPα-Hsc70, synaptotagmin with complexins, and VAMP2 with α-synuclein). Multiple layers of regulation are present for each component, with both synaptotagmin and α-synuclein being functionally calcium-dependent [64,65,66]. The requirement for α-synuclein to embed into lipid vesicles is not a primary target of regulation as it is the disequilibrium between lipid-bound and monomeric species that may propagate disease pathogenesis. In addition to the above function, α-synuclein modulates synaptic plasticity through enhancing dopamine reuptake from the presynaptic cleft. α-Synuclein-knockout mice exhibit minimal dysfunction in terms of neurotransmission but do have altered dopamine reuptake in the nigrostriatal pathway [67]. However, when silencing all three synuclein isoforms (α, β, and γ), mice exhibit significantly impaired membrane fusion and SNARE complex formation [38]. Silencing all synuclein isoforms spawned age-dependent dysfunction in vivo and in vitro, evidently mediating synaptic junctions to diminish by ~30% in size, retinal degeneration, increased VAMP2 expression, and poor survivability [68].

After exocytosis, α-synuclein modulates synaptic vesicle recycling and subsequently docks at dopamine transporters (DAT) via weak *C*-terminal interactions [69,70]. It remains unclear exactly how dopamine reuptake occurs, but docked vesicles bind DAT through the transmembrane protein synaptogyrin-3, facilitating dopamine–proton exchange [71]. As a result, dopamine reuptake is trafficked directly into synaptic vesicles, avoiding oxidative cytotoxicity. In addition to its synaptic role, α-synuclein may also participate in endoplasmic reticulum (ER) to *trans*-Golgi network (TGN) trafficking by interacting with RAS-related protein in brain 1a (Rab1a) [72]. The latter GTPase has been linked to macroautophagy and autophagosome trafficking, as shown by its overexpression rescuing cells from α-synuclein-dependent dysfunction of ferritinophagy in transfected retinal pigmented epithelial cells [73].

## 4. Iron and Calcium Regulation of α-Synuclein

Regulation of α-synuclein is almost entirely at the post-transcriptional level, depending upon numerous metal ions for functional activity. Up to eight calcium ions bind along the *C*-terminus at residues E104, A107, I112, D119, E123, A124, and E126 [37]. However, physiological scenarios only require six calcium atoms for functionality. In vitro, α-synuclein monomers can only interact with hydrophobic surfaces at its *N*-terminus, yet, when calcium was added to the suspension, hydrophobic interactions with phospholipid membranes were increased by 5-fold [48]. Calcium enhanced the hydrophobicity across the *C*-terminus and central non-amyloid component (NAC) domain, promoting membrane interaction [74].

### 4.1. Aggregation Induction of α-Synuclein

In vivo, voltage-gated calcium channels (VGCCs) can increase calcium concentrations to 200–300 µM in microdomains, exceeding the *K_d_* of 21 µM for α-synuclein [37,75]. Furthermore, copper ions, bound at D2 and H50, support the folding of α-synuclein *N*-terminal helices [76]. Unlike other metals, the interactions between iron and α-synuclein are far less understood. On the one hand, reports have described α-synuclein as a ferrireductase, catalyzing the reduction of Fe^3+^ to Fe^2+^ [77]. While on the other hand, nuclear magnetic resonance (NMR) spectroscopy suggests that iron has a poor micromolar affinity and may only interact with the Asp121 residue [78,79].

Fibrillogenesis (i.e., the generation of amyloid-like fibril structures) stems from calcium exposure to monomeric α-synuclein, ultimately prompting hydrophobic interactions between monomeric species through β-sheet stacking [37,80]. Notably, the enhanced hydrophobicity amongst the NAC domain is the driving force behind fibril development [61,81]. However, the complete misfolding of monomeric α-synuclein can yield annular species that may lodge in lipid membranes and distort cellular permeability to ions [82]. In the context of toxicity, preformed fibrils can substantially impede cell viability at nanomolar concentrations, despite no endogenous expression of α-synuclein in certain cell types [10,83,84]. It has been suggested that the *SNCA gene* contains an atypical iron response element (IRE) in its 3′-untranslated region (3′-UTR) [85]. Such a theory could indicate that intracellular iron concentrations may promote α-synuclein expression. However, it is essential to note that this IRE may be non-functional, as it does not conform to typical canonical sequences that bind iron-response proteins (IRPs) [86]. Further studies are required to determine if this IRE plays any role in the regulation of α-synuclein expression by iron.

### 4.2. Iron-Induced Catecholamine Oxidation and Redox Damage

**Figure 2:**
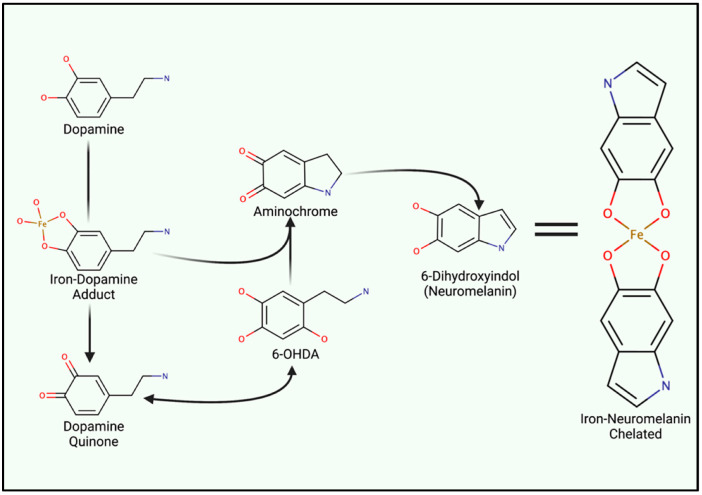
Iron Involvement in Dopamine Oxidation and Neuromelanin synthesis. The hydroxyl groups of dopamine can directly chelate iron, catalyzing the generation of either aminochrome or dopamine quinone. Both analogues induce mitochondrial dysfunction and oxidative stress. However, quinone species can undergo reactions to generate 6-hydroxydopamine (6-OHDA). Only differing by an additional hydroxyl group, 6-OHDA can enter cells through DAT to induce internal redox stress. Acidic vesicles provide a reducing environment for aminochrome carbonyl groups, ultimately yielding 6-dihydroindol (neuromelanin) which can form adducts between reactive ions.

Iron metabolism is important when considering the marked redox potential of catecholamines. For example, iron can ligate to the adjacent hydroxyl groups of dopamine [6,87] (Figure 2). The resultant dopamine quinone can be further transformed into 6-hydroxy dopamine (6-OHDA) in the presence of hydrogen peroxide (H_2_O_2_) [88]. 6-OHDA is a potent neurotoxin used to replicate PD pathologies in mice, and unlike 1-methyl-4-phenyl-1,2,3,6-tetrahydropyridine (MPTP), 6-OHDA specifically damages dopaminergic neurons through DAT-mediated uptake [88]. Aminochrome synthesis may occur directly from iron–dopamine adducts or as a downstream 6-OHDA product. At physiological pH, aminochrome is cytotoxic through redox cycling. The subsequent production of 6-dihydroxindol (neuromelanin) is neuroprotective, presumably via the chelation of redox-active iron [89]. Ultimately, neuromelanin seemingly suppresses redox cycling in acidic vesicles (e.g., synaptic vesicles, autophagosomes, and lysosomes) [88].

Neuromelanin chelates iron and other reactive products via two hydroxyl groups upon the fifth and sixth carbon (Figure 2), yielding a benzothiazepine-like complex [8,88,90]. Redox-active iron and copper can directly oxidize dopamine; thus, both can induce neuromelanin synthesis and chelation. However, iron is by far the most prominent substrate [91]. Isolated neuromelanin contains ~11 μg/mg of iron, while basal tissue concentrations remain approximately 0.1–0.25 μg/mg [89,92]. Neuromelanin localizes to autophagosomes due to its autophagy-dependent clearance (melanophagy) [93]. Neuromelanin concentrations increase with age and in response to prolonged exposure to reactive species and lysosomal failure, giving rise to age-dependent pigmentation. Transmission electron microscopy of healthy *substantia nigra* and *locus coeruleus* tissue illustrates neuromelanin-positive autophagosomes that exceed 1 µm in diameter [93]. Vesicular clustering, increased metal concentrations, and autophagy-lysosomal dysfunction are both indicative of senescent cells as well as many neurodegenerative diseases (e.g., Alzheimer’s disease (AD), HD, and amyotrophic lateral sclerosis (ALS) and PD) [94,95,96,97,98].

## 5. Iron Entry, Regulation and Cellular Metabolism

Iron acquisition by cells can be separated into transferrin-dependent or transferrin-receptor-independent mechanisms. The transferrin receptor is not expressed in glia, yet these cells become burdened with high intracellular iron levels and ferritin in disease states [99,100]. Moreover, the CNS contains a high concentration of low molecular weight iron with some proportion being chelated and reduced to Fe^2+^ by ascorbate [101,102]. The mechanism of how iron loading occurs in dopaminergic neurons remains elusive. In recent years, VGCCs, particularly those of the L-type family, has been recognized as being permeable to iron (Fe^2+^), thus providing a potential path for unregulated iron entry and intrinsic redox damage [103].

## 6. Iron Metabolism and Ferritin

**Figure 3:**
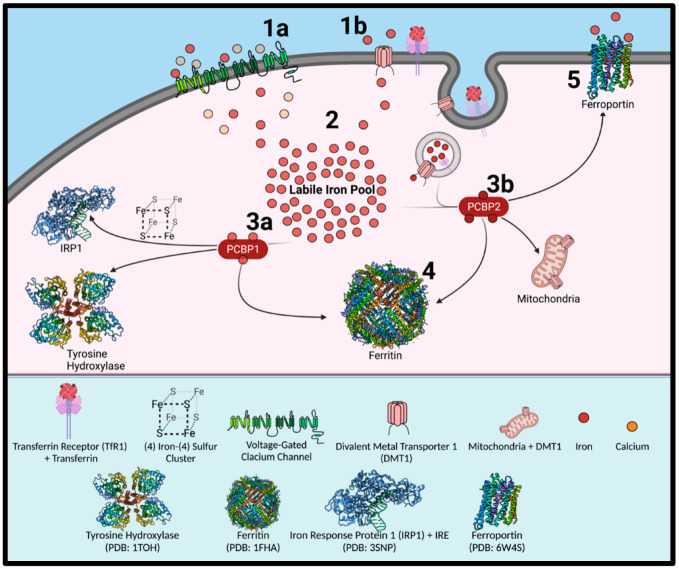
Mechanisms of Iron Uptake and Intracellular Trafficking. Iron influx through transferrin-bound or non-transferrin-bound pathways (1), with the latter including potentially unregulated entry through voltage-gated calcium channels in dopaminergic neurons (1a). Transferrin receptor 1 (TFR1) and DMT1 (1b) and the cytosolic iron chaperones, poly-r(C)-binding proteins (PCBPs), play key roles in iron uptake and intracellular trafficking. High levels of iron in the labile iron pool leads to its storage in ferritin (2). PCBP1 participates (3a) in iron delivery to iron sulfur clusters (4Fe:4S) for proteins such as iron regulatory protein 1 (IRP1), while also directly trafficking iron to enzyme active sites. PCBP2 regulates intracellular iron concentrations by chaperoning the metal towards the iron efflux pump, ferroportin (3b). PCBP2 also chaperones iron after its transport across the endosomal membrane, with this occurring by its direct binding to DMT1 binding. All known PCBPs (1–4) can deliver iron to ferritin nanocages (4). Ferritin releases its stored iron by the process of ferritinophagy. Made with BioRender.com.

There are many attributes that overlap autophagy and endocytosis [104], meaning that molecular similarities may exist between transferrin receptor 1 (TFR1) regulation (receptor-mediated endocytosis) and ferritin regulation (ferritinophagy) (Figure 3). This is particularly true as autophagosomes and endosomes can fuse (creating an amphisome) for extracellular release [105,106]. Once transferrin binds the TfR1, endocytosis occurs leading to an endosome containing the transferrin-TfR1 complex. Once iron is released from transferrin after a decrease in endosome pH, the ferrireductase, six transmembrane epithelial antigen of the prostate 3 (STEAP3), converts Fe^3+^ to Fe^2+^ for transport across the endosomal membrane by the divalent metal ion transporter 1 (DMT1) [107,108].

Previous evidence has suggested potential involvement of DMT1 in PD pathogenesis. However, DMT1 is stringently regulated at multiple levels [109,110,111]. The DMT1 mRNA population is heterogeneous, with two alternative transcripts that differ at their 3′-UTR by either containing an IRE (+IRE; Type 1) or lacking an IRE (−IRE; Type 2) [112]. DMT1 functionality is poor at pH 7, requiring an acidic environment of pH 5.5–6.5 for transport activity [110,113]. After iron is transported by DMT1 across the endosomal membrane, it then binds to the iron chaperone, poly-r(C)-binding protein 2 (PCBP2) [114,115,116]. Direct binding of PCBP2 to the cytosolic face of DMT1 facilitates iron transport, with the PCBPs forming part of the intracellular labile iron pool (LIP) [117]. PCBP2 functionally traffics iron to cellular compartments, including the iron efflux pump, ferroportin (FPN1) [116]. PCBP2 also associates with heme oxygenase to remove iron from heme breakdown sites [118]. PCBP1 delivers iron to the active sites of multiple iron-containing enzymes [115,117], with PCBP1 acting together with PCBP2 as a co-chaperone to deliver iron to acireductone dioxygenase [119]. Furthermore, as part of their critical roles in iron metabolism, PCBPs play an essential role in chaperoning iron to ferritin for storage [117].

**Figure 4:**
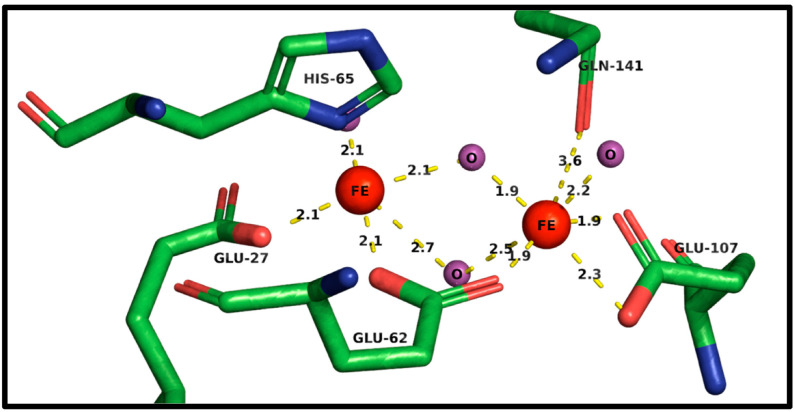
Ferritin Heavy Chain (FtH1) Ferroxidase Active Site for Iron Entry into the Ferritin Cage. The FtH1 ferroxidase active site. Ferrous (Fe^2+^) iron enters through the pore-like domains and is delivered to glutamate (Glu) 27 and then to Glu62. Histidine (His) 65 prevents back flow and transfers iron towards the carboxyl group of Glu62. Ferrous iron is then converted into ferric (Fe^3+^) iron passing via Glu62, Glu107, and glutamine (Gln) 141. All bond lengths are in angstroms (Å). Green chains represent amino acid carbon backbone structures, red represents oxygen groups, and blue represents nitrogen groups. Red FEE) orbs infer iron atoms while purple (O) infers free oxygen ions. Created using PyMOL2 and RCSB PDB: 1FHA.

Ferritin is a dedicated iron storage protein complex consisting of a heavy chain (FtH1) and a light chain (FtL) [14,120]. Storage of up to 4500 Fe^3+^ ions within its 8 nm internal diameter cage can be a cytoprotective strategy to minimize the labile iron pool (LIP) [18,120]. In the liver and spleen, the subunit ratio of ferritin heavy chain 1 (FtH1) and ferritin light chain (FtL) is 1:1. Yet, within the brain and heart, the ratio is variably offset in favor of FtH1 [121]. PCBPs deliver iron to FtH1 for incorporation, with FtL homodimers unable to efficiently bind the chaperones [115,116]. Structurally, the ferritin subunits form tetra-barrel pores, where FtH1 can facilitate iron import while FtL cannot. Iron can pass through the structural pores via glutamate residues where Fe^2+^ atoms bind to the sidechains of Glu27 and Glu63 (Figure 4), while His66 prevents backflow. Gln142 serves as an electron acceptor for oxidation of iron into Fe^3+^ [121]. The additional valency permits Glu108 binding and subsequently, the internalization of iron atoms. Ferroxidase activity is low in FtL subunits [122]. It is likely that ferrous iron can permeate and undergoes oxidation, possibly via the mineralized ferric hydrate core [121].

Mitochondrial ferritin (FtMt) is another ferritin superfamily relative and shares >80% homology with FtH1 [123]. Inheriting the ferroxidase ability, FtMt specifically localizes to the inner mitochondrial membranes due to an *N*-terminally located mitochondrial targeting sequence that is proteolytically cleaved upon entry. In differentiated SH-SY5Y neuroblastoma cells, the overexpression of FtMt significantly reduced α-synuclein by 35% at the post-transcriptional level but not at the mRNA level [124]. Furthermore, Western blotting found that 100 μM FeCl_3_ significantly increased α-synuclein level, and again, this result was not consistent with mRNA expression. Treatment with 100 μM H_2_O_2_ caused increased FtMt expression, likely as a protective response against mitochondrial oxidative stress [124]. In 80 μM H_2_O_2_ treatment groups, FtMt overexpression significantly reduced α-synuclein concentrations [124,125,126].

It is not currently understood how FtMt can translocate across the mitochondrial membrane, but one possible scenario would be that protein import occurs via the mitochondrial import receptor subunit translocase of the outer membrane of 20 kDa homolog (TOMM20) complex. Oxidative phosphorylation and excessive proton exchange generates a high positive charge across the inner mitochondrial membrane (IMM), a feature that attracts the lipophilic *N*-terminus of α-synuclein. In vivo, non-pathogenic α-synuclein species can be embedded in the inner and outer mitochondrial membrane [127,128]. With a low molecular weight of 14 kDa, α-synuclein is small enough to translocate into the IMM via the TOMM20 complex, suggesting that α-synuclein may have a role in mitochondrial function, possibly during membrane fusion events. Upon fibrilization, the electrophilic *N*-termini can initiate translocation across TOMM20 complexes but block the import receptor due to the aggregated NAC domain [129,130], attenuating protein import.

## 7. Autophagy and Ferritinophagy

Autophagy is a lysosomal degradation pathway that regulates many cellular events such as mitochondrial health and protein disposal and historically plays an integral role in nutrient availability upon amino acid starvation [131,132]. Autophagy is subdivided into three tiers based upon cargo size. Microautophagy is where small organelles and insoluble products such as peroxisomes and lipid droplets are directly trafficked across lysosomal membranes [131]. Chaperone-mediated autophagy (CMA) utilizes heat shock proteins (Hsp) as cargo chaperones for the trafficking of KFERQ motif-containing proteins towards lysosomal-associated membrane protein 2a (LAMP2a) [59].

Misfolded α-synuclein monomers are CMA targets sequestered to cytoplasmic Q-bodies, becoming refolded by Hsc70-Hsp90-Hsp40-HOPS (homotypic fusion and vacuole protein sorting) complexes [133]. The unsuccessful refolding of α-synuclein inhibits the ubiquitin-proteasome system (UPS) degradation and CMA [104,134,135]. Both UPS and CMA require cargo peptides to be unfolded prior to degradation. However, the extensive hydrophobic binding and cross-linking of α-synuclein aggregates prevent efficient reorganization, thus halting degradative attempts. Much like the TOMM20 complex, LAMP2a becomes blocked by oligomeric α-synuclein species that are unable to be chaperoned by Hsp90 into the lysosomal lumen [136,137,138]. Macroautophagy is the highest order autophagy subtype, involving the dynamic formation of a double membrane vesicle around cytosolic cargo [94,131,139]. From here on, macroautophagy will be referred to as autophagy as it is the primary focus within the current review. Cellular stress, such as amino acid starvation [131] inhibits the serine/threonine kinase mammalian target of rapamycin (mTOR) [140]. Normally, mTOR complex 1 (mTORC1) suppresses the two most upstream autophagy regulators, AMP protein kinase (AMPK) and Unc-51 like autophagy activating kinase (ULK1), by directly phosphorylating them both [141,142,143].

**Figure 5:**
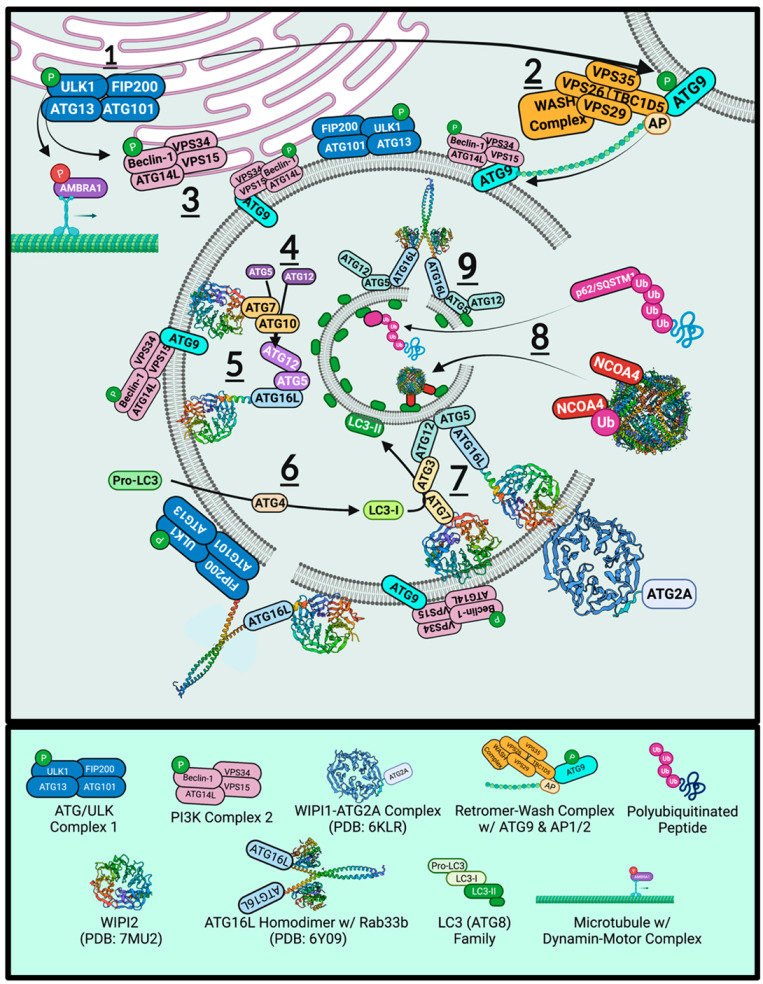
Autophagy Signaling, Phagophore Biogenesis, and Autophagosome Maturation. The phosphorylation of ULK1 assembles the ULK1/ATG complex (1) which in turn, phosphorylates ATG9 (2) to promote retromer trafficking to omegasomes and (3) Beclin-1 for Class III PI3K complex construction. ULK1-dependent phosphorylation of AMBRA1 is inhibitory and releases the Class III PI3K complex upon omegasome arrival. WIPI2 houses two subsequent complexes that form on the first internal phagophore surface. The first involves (4) the ATG7–ATG10 dimer that exhibits E1–E2 mechanisms to covalently conjugate ATG12 and ATG5, binding ATG16L afterwards (5) for a structural scaffold. Cytosolic Pro-LC3 (ATG8) is *C*-terminally cleaved by the ATG4 protease to yield LC3-I (6). It is then prenylated to internal PE segments for receptor-cargo delivery (8). Once saturated, ATG16L can form homodimer complexes (9) to constrict segments for closure. ATG16L may also form coiled-coil heterodimers with FIP200 on the external surface to facilitate outer membrane closure.

Increased adenosine monophosphate (AMP) primes AMPK to phosphorylate ULK1 (Figure 5, part 1). Depending upon the stimuli, specific and non-specific mechanisms of autophagy can be induced, where non-specific autophagy involves the engulfment and degradation of random cytoplasmic contents for rapid amino acid recycling [131]. Whereas, specific autophagy is highly coordinated and requires adaptor proteins to directly traffic protein aggregates (aggrephagy) [144,145], dysfunctional mitochondria (mitophagy) [128,146], ferritin (ferritinophagy) [19,20,147], bacteria (xenophagy) [148], and other cytosolic components trafficked towards autophagosomes. The following subsections cover the specific components of autophagy, relating the topic to ferritin and PD pathogenesis.

### 7.1. ULK1

ULK1 has two globular domains termed the *N*- and *C*-lobes, each with separate functions [149]. The *N*-terminal lobe includes a kinase domain, binding ATP centrally between the two lobes. Here, a P-loop structure houses the ATP binding sequence of G(X)G(X) FAA (where X is any residue) [106]. Active ULK1 forms complexes with ATG13 (autophagy-related protein 13), ATG101 (autophagy-related protein 101), and FIP200 (FAK family kinase-interacting protein of 200 kDa; or RB1-inducible coiled-coil protein 1, RB1CC1) to form the ATG/ULK complex [143,150]. Activated ULK1 then phosphorylates beclin-1 [141,151] (Figure 5, part 3), GABARAP (ATG8 γ-aminobutyric acid receptor-associated protein), and SYNGAP (the synaptic Ras-like GTPase-activating protein 1) to stimulate endoplasmic reticulum remodelling for autophagosome formation. ULK1-dependent phosphorylation of p62/SQSTM1 (p62/Sequestosome-1) and ATG9 (autophagy-related protein 9) also assist in pathway progression (Figure 5, part 2) [143,152,153]. Downstream of ULK1, the retromer complex actively enriches endoplasmic reticulum sites with phosphatidylinositol 3-phosphate (PI3P), yielding phagophore initiation sites termed omegasomes [104].

### 7.2. Retromer

The retromer complex is a master trafficker of endocytic vesicles and *trans*-Golgi network (TGN) content, being primarily composed of vacuolar protein sorting-associated protein 26A (VPS26), VPS35 and VPS29 [154,155,156,157,158]. The complex can also recruit the Rab-activating protein TBC1 domain family member 5 (TBC1D5), which is required for Ras-related protein Rab-7a (Rab7)-dependent events such as mitophagy and late endosomes [159,160]. In endosomal sorting, a loss-of-function D620N mutation in VPS35 showed increased DMT1 levels due to failed lysosomal targeting [157,158,161,162]. Instead, DMT1-II colocalized with LAMP2A structures on lysosomal membranes, suggesting that DMT1 might be enhancing the LIP through the efflux of lysosomal iron in VPS35 D620N mutant pathogenesis. The retromer is also involved in lysosomal biogenesis by sorting the cation-independent-mannose-6-phosphate receptor (CI-MPR), involved in lysosomal acidification [135]. Thus, VPS35 mutations, also known as the *PARK17* locus, can cause classical PD with autosomal dominant inheritance [163]. Furthermore, developing autophagosomes require the retromer complex orchestrated with the Wiskott–Aldrich syndrome protein and SCAR homologue (WASH) complex, which contains the seven-subunit actin-related proteins 2/3 (Arp2/3) for actin nucleation [161,164,165].

### 7.3. ATG9

ATG9 is a retromer-WASH complex substrate that provides the scaffolding for autophagosome development [166]. As the only transmembrane autophagy protein, ATG9 localizes to lipid membrane sources and is phosphorylated at S14 by ULK1 [167]. Phosphorylation permits adaptor protein 1/2 (AP1/2) binding and retromer complex binding via TBC1D5 (Figure 5, part 2) for trafficking towards omegasomes [160]. ATG9 and lipid segments are extracted from plasma membranes and used to construct autophagosomes. Actin microtubule nucleation via the WASH complex means that membrane segments can dynamically be delivered to developing autophagosome sites, even after detachment from the ER. ATG9 can be readily exported to a proximal membrane reservoir during phagophore mobilisation, such as mitochondria, multivesicular bodies (MVB), plasma membranes, ER, and Golgi [106,168,169]. Dispersed ATG9-positive PI3P regions can also act as phagophore development sites, independent of omegasomes. ATG9 KOs significantly reduce autophagosome size and quantity in drosophila models [105].

Depleted cells lack Rab11 intraluminal vesicles and have aberrant acidification of amphisomes (fused autophagosome–late endosome structures targeted for extracellular release). The release of amphisomes stem from SNAP23 interactions at the plasma membrane, notably if the t-SNARE is phosphorylated at S95 and S110 [105,170,171]. Winslow et al., (2010) reported that ATG9 colocalizes to the TGN but can be detected throughout the cytoplasm, where it colocalizes with the autophagosome marker LC3-II [172]. Overexpression of α-synuclein or Rab1a KO models exhibited significantly diminished ATG9 colocalization with ER membranes and autophagosomes, indicating that α-synuclein directly inhibits ATG9-derived omegasomes and autophagy, particularly through inducing ER fragmentation.

### 7.4. Beclin-1 and Vps34

Beclin-1 is the principal ULK1 substrate, the structural backbone of the phosphatidylinositol 3-kinase catalytic subunit type 3 (PI3KC3-C1) lipid kinase complex [141,149,151]. Menon and Dhamija (2018) have reviewed beclin-1 phosphorylation recently, detailing how ULK1-dependent modification influences autophagy signalling [153]. Beclin1 contains a BH3-domain that is subject to phosphorylation by RAC-alpha serine/threonine-protein kinase (AKT1) (S234 and S295), causing apoptosis regulator B-cell lymphoma 2 (BCL-2) or B-cell lymphoma-extra-large (BCL-XL) conjugation and inhibiting interactions. Furthermore, phosphorylation of the *N*-terminal residues by ULK1 (S15 and S30) or AMPK (S93, S96, and T388) frees beclin-1 from BCL2, so that the central coiled-coil domain (CCD) can bind the autophagy-related ATG14L or UV radiation resistance-associated gene protein (UVRAG).

The *C*-terminus of ATG9 is highly conserved, possessing a β/α-repeated autophagy-related domain (ECD-BARA domain collectively) that ligate Vps34 and Vps15. Autophagy and beclin-1 regulator 1 (AMBRA1) anchors beclin-1-Vps34-Vps15 to dynamin light chains for microtubule-dependent transport to omegasomes. ULK1, localized to ER omegasomes, phosphorylate AMBRA1 to mediate cargo detachment from the dynamin transporter. Vps15 enhances the class III PI3K Vps34 via Rab5 to give PI3P, thus anchoring the complex to ER membranes and spawning omegasomes.

### 7.5. WIPIs and ATGs

Downstream of PI3KC3-C1 consists of a three-core ubiquitin-like E1, and E2 enzyme complex. The effector protein WD repeat domain phosphoinositide-interacting protein 2 (WIPI2) assumes a β-propeller structure on phagophore membranes [173,174]. Neurodegeneration with brain iron accumulation 5 (NBIA5) is an X-linked genetic disorder that harbours a mutation within WIPI4 [175]. NBIA5 causes static cognitive development in early childhood and neurodegeneration in early adulthood [176]. In addition, affected individuals develop dystonia, dementia-like cognitive impairment, and parkinsonisms. Furthermore, magnetic resonance imaging (MRI) detects iron accumulation within the *globus pallidus* and *substantia nigra*. Moreover, WIPI localizes to autophagosomes and participates in transferring lipid membrane segments, particularly at the autophagosome-lysosome fusion [177].

WIPI2 contains ATG7–ATG10 (Figure 5, part 4), which acts as an E1–E2 system that covalently links ATG12–ATG5 (Figure 5, part 5) [141,174,178]. In parallel, the ATG8 family member of microtubule-associated proteins 1A/1B light chain 3 (LC3) is lipid-conjugated (Figure 5, part 6) [179]. ATG4B is the cysteine protease that cleaves the cytosolic pro-LC3 *C*-terminus, exposing the G120 residue (LC3-I) [180]. The glycine residue is then covalently prenylated to the phosphatidylethanolamines (PE) amino group by ATG7-ATG3 (Figure 5, part 7). Again, the duo acts as an Ub-like E1–E2 complex, yielding LC3-II-linked PE strands. After fragmented components have been synthesized and ATG9 segments have been delivered, the membrane pieces are clustered into a functional autophagosome.

### 7.6. ATG2 Tethering and ATG16L1 Constriction

ATG16L1 non-covalently binds ATG12–ATG5, enhancing LC3-II lipidation in vivo and in vitro [177,178]. ATG12 conjugates to ATG3, facilitating LC3 lipidation to PE. Yet, the primary role of ATG16L-ATG5-ATG12 complexes involves the coiled-coil domain of ATG16L (Figure 5, part 9). WIPI2 recruits ATG16L and forms a coiled-coil while the latter binds ATG5. Direct lipid interaction between ATG5 and PE allows ATG12 to bind ATG3 and prenylated LC3-II. The coiled-coil ATG16L–WIPI2 structure may detach as a homodimer complex that constricts, joining two separate PE segments. Additionally, ATG2A tethers developing autophagosomes to various membranes and complexes with WIPI4 and WIPI1 to facilitate lipid transfer between membranes [181]. However, ATG16L exhibits a multitude of roles in phagophore biogenesis. Reports have suggested that ATG9 segments may exclusively fuse with ATG16L-positive vesicles.

### 7.7. ATG8

There is a single ATG8 protein in yeast, whereas mammalian cell types require a family of ATG8 proteins, including LC3 (LC3a, LC3b, LC3c), GABARAP, and γ-aminobutyric acid receptor-associated protein-like 1 and 2 (GABARAPL1 and GABARAPL2). LC3s are essential for phagophore development (Figure 5, part 7). The most noticeable disparity between isoforms is that LC3a predominantly participates in microtubule trafficking. Its homologue LC3b has been suggested to participate in the assembly of microtubules, particularly during neurogenesis. LC3b is the central protein binding autophagic cargo to the vesicular membrane. Furthermore, LC3s influence membrane curvature in ~10 nm segments [182]. Concave architecture is then further accentuated by ATG3 via its 20-residue amphipathic *N*-terminal α-helix. As LC3 is prenylated and attached to an intraluminal PE membrane, numerous partners can interact in structural or degradative roles.

The LC3 interacting region (LIR) is a canonical φ_1_(W/F/Y)-X_2_-X_3_-φ_4_(L/I/V) motif (where X is any acidic, basic, or hydrophobic residue) that is flanked by at least one acidic residue and is required for LC3-substrate activity. LIRs can be grouped by their ϕ_1_ residue (W-type, F-type, Y-type), where W-type (tryptophan) provides the highest affinity for the hydrophobic pocket 1 (HP1) site in LC3 and uniquely incorporates K51 and F108. Recent studies [180] suggest that Y-type motifs are cargo-receptors. Tyrosine (Y) residues can participate in redox reactions which may be relevant to a potential role of Y-type LIR activation in oxidative stress. LC3 and GABARAP are covalently bound to PE membranes. Because of this, LC3 is widely used as a primary marker for autophagosomes and autophagic flux in immunofluorescence experiments. Co-localization experiments between a target protein and LC3 provided evidence of the autophagosomes engulfing cytosolic cargo, particularly in response to external stimuli. An assessment of mammalian ATG8 orthologues in the spinal cord and cortical tissue from PD and dementia with Lewy bodies (DLB) patients found that ~40% of LBs were immune-positive for LC3-II [183], whereas only ~15% stained positive for GABARAP/GABARAPL1. Compared to controls, patient tissues exhibited a 76% reduction in GABARAP concentrations, whereas no significant association was observed for LC3. However, examination of cellular fractions determined that DLB tissue had significantly higher levels of insoluble LC3 (LC3-I and LC3-II). These findings indicate that primary disruption occurs downstream of LC3 lipidation.

### 7.8. NCOA4 and LC3-Interacting Region

Ferritinophagy utilizes the specialized adaptor protein nuclear co-activator 4 (NCOA4) to bind ferritin via ubiquitination or directly by FtH in an iron-dependent manner (Figure 5, part 7). Functionally, autophagy cargo-receptors chaperone cytosolic cargo towards developing autophagosomes, binding to prenylated LC3. Binding generates a membrane-LC3-adaptor-cargo linkage. Chaperones require internal LIRs to complete cargo linkage. To date, no publication has identified the LIR within NCOA4. In Table 1*,* two potential regions that conform to the LIR φ_1_-X_2_-X_3_-φ_4_ motif stipulation, including the flanking acidic residue, are shown. No W-type motifs could be identified in the NCOA4 transcript. It has been hypothesized previously that adaptor and regulatory proteins are likely to contain Y-type motifs, which is supported as Table 1 identifies a multitude of Y-type and F-type homologous motifs in NCOA4 [180]. NCOA4 is regulated in an iron-dependent manner by HECT and RLD domain containing E3 ubiquitin protein ligase 2 (HERC2). In iron-enriched environments, the HERC2 Ub-E3 ligase ubiquitinates NCOA4 at lysine residues and targets the adaptor protein for UPS degradation. Iron deprivation inhibits HERC2, enhancing NCOA4 stability.

### 7.9. p62/Sequestosome-1

The cargo-specific adaptor protein p62 (also known as sequestosome-1; SQSTM1) is implicated in most autophagy pathways (e.g., aggrephagy and mitophagy) (Figure 5, part 7). ULK1 phosphorylation of p62 does not occur under canonical nutrient deprived autophagy activation [143]. Instead, proteasomal inhibition prompts phosphorylation at S409, promoting a shift towards autophagy-dependent clearance [184]. Phospho-S409 p62 also increases in response to poly-Q-huntingtin (Htt), with the expression level correlating with the length of poly-Q-repeats [152]. Phospho-S409 p62 has a significantly increased affinity for ubiquitinated cargo. p62 KO models exhibit no alteration in the autophagosome biogenesis, instead, aggresomes fail to be correctly trafficked towards the encapsulating vesicles [185]. One report by Nihira et al. (2014) suggests that p62 KOs increase LC3-II conversion. Justifying this, cell stress would induce the formation of LC3-II, yet a lack of p62 would mean that cargo fails to be delivered to autophagophores [186]. This multi-potent adaptor protein, p62, has been well characterized. However, here we will focus upon the role of p62 in chaperoning cargo.

Ubiquitin binding occurs at residues 389–434 via an ubiquitin-associated (UBA) domain, while p62 also contains a downstream LIR motif (residues 336–341) for attachment to autophagosome membranes [187,188]. Both domains are *C*-terminally located, leaving the *N*-terminus free to interact with a variety of other targets, such as γ-aminobutyric acid receptor subunit rho-3 (GABRR3) (residues 122–224) and PRKC-apoptosis-WT1-regulator (PAWR) (residues 50–80) [189]. The latter has been identified as a positive regulator of β-secretase 1 in amyloid precursor protein (APP) cleavage [190]. Furthermore, several point mutations in the p62 gene have been reported that are related to frontotemporal lobar dementia (FTLD) and ALS, some of which alter adaptor binding to poly-ubiquitinated cargo [191]. Transcriptional control of p62 is through nuclear factor erythroid 2–related factor 2 (Nrf2), a focal antioxidant responsive transcription factor, which is diminished in PD tissue and cell models subject to α-synuclein preformed fibrils [126,192].

### 7.10. SNARE Proteins

Various studies have suggested that SNAREs, ATG proteins, and LC3-lipidation may all participate in membrane elongation. SNARE proteins can be broken down into two groups: vesicular (v)-SNAREs and target (t)-SNAREs. The vesicular group all contain R-SNARE motifs, while t-SNAREs are segregated by their internal motifs Qa-SNARE (syntaxin family), Qb-SNARE (*N*-terminal SNAP25), and Qc-SNARE (*C*-terminal SNAP25). Significant functional redundancy exists amongst SNARE proteins as complexes require all components (R-SNARE and Qabc-SNARE motif) to generate a trans-SNARE complex that will mediate membrane fusion successfully. The canonical SNARE complex involved in autolysosome fusion is a VAMP7/8-syntaxin-17/STX17-SNAP29 complex [179]. ATG14 contains a tandem transmembrane glycine-zipper-like motif and can complex with syntaxin-17 [193,194]. ATG14L-STX17-SNAP29 complexes prime VAMP7/8 fusion. ATG14 and syntaxin-17 can also recruit homotypic fusion and vacuole protein sorting (HOPS) complex and facilitate STX17 docking at ATG9-positive sites for phospholipid tethering [195]. In pancreatic beta-islet cells [196], VAMP7 resides in ATG9a-positive vesicles along with Rab11 [197]. The latter primarily localizes to slow recycling endosomes but can reside in other endosomal recycling compartments. KO studies of the HIV-1 Rev-binding protein (HRB) resulted in significant protein trafficking impairments. Notably, HRB-KO induced VAMP7 and ATG9a colocalization at the plasma membrane, thus reducing the proximal ATG9-positive vesicle pool.

The functional role of syntaxin-16 overlaps with synataxin-17. However, there is one prominent dissimilarity. Syntaxin-16 contains the important LIR motif and has proven critical in retrograde endosomal-Golgi trafficking [179,198,199]. Taken together, VAMP7-syntaxin-16-SNAP47 likely collaborates at a functional SNARE complex that is implicated in autophagosome biogenesis. Furthermore, KO models of syntaxin-7, syntaxin-8, and VAMP7 all exert little effect on phagophore biogenesis until the ATG16L mutation is incorporated. Upon SNARE protein knockout, ATG16L-positive clusters accumulate at omegasomes [150,200,201]. At the autolysosome level, silencing of syntaxin-16 or syntaxin-17 causes a minimal alteration to pathway phenotype as these isoforms share functional redundancy. However, silencing abolishes all autolysosome fusion events and exhibits phenotype-like chloroquine-treated cultures.

## 8. α-Synuclein Aggregates Inhibit Ferritinophagy

As α-synuclein aggregates impede vesicular trafficking [202,203], multiple cellular systems are burdened with immense stress. Ferritinophagy maintains ferritin turnover, which is essential when considering the large amount of reactive iron held within, particular for iron-loaded nigral neurons [21]. Recent evidence suggests that ferritin is encapsulated within autophagosomes as α-synuclein potently inhibits autolysosome fusion while less potently altering phagophore biogenesis [73]. As α-synuclein alters autophagy and vesicular trafficking events at the membrane fusion stage, little effect would be expected on NCOA4-dependent ferritin trafficking towards autophagosomes. Thus, ferritin would be expected to remain within the double membrane structures until successfully degraded or sufficiently trafficked towards removal.

**Figure 6:**
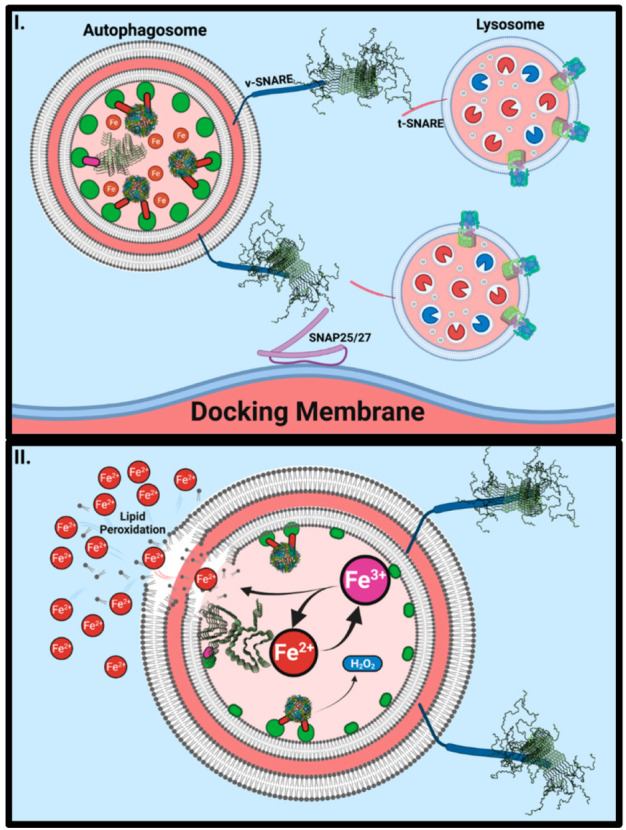
α-Synuclein may Inhibit Ferritinophagy by Blocking Autolysosome Fusion. Aggregated α-synuclein (PDB: 2N0A; 6FLT) binds to and blocks the function of v-SNAREs (**I**) such as VAMP2 in the case of synaptic function and VAMP7 and VAMP8 in the case of autophagy. Consequently, lysosomes are unable to fuse with engorged autophagosomes to facilitate the retrieval of iron from ferritin storage. As ferritin starts to breakdown within acidic vesicles, ferric iron (Fe^3+^) will be released within the vesicle where reduction can cause lipid peroxidation (**II**). Hydrogen peroxide is contained within ferritin (PDB: 1FHA) and will also be released upon denaturation. H_2_O_2_ will utilize ferrous iron as an electron donor for reduction events, potentially giving rise to a positive feedback loop within a vesicle that cannot be removed.

**Figure 7:**
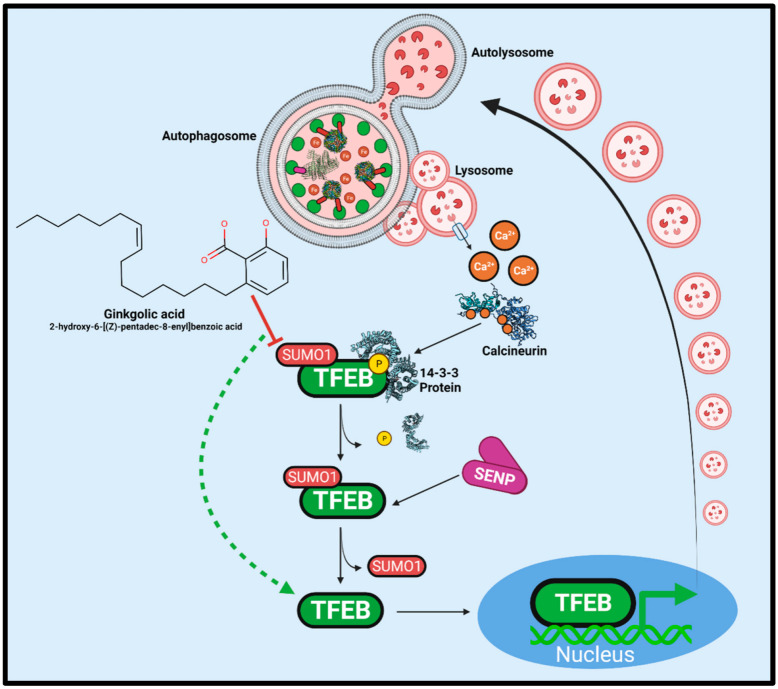
TFEB Regulation by Calcineurin and SUMO1-modification. Accumulated autophagosomes and the resultant lysosomal stress causes increases in cytosolic calcium concentrations. Calcium ions bind to activate the serine/threonine phosphatase calcineurin (PDB: 4OR9). Reversing TFEB phosphorylation causes the dissociation of the inhibitory 14-3-3 (PDB: 6A5Q) family of proteins to enhance nuclear translocation. However, conjugated SUMO1 prevents nuclear pore transport and requires cleavage by a sentrin-specific protease (SENP). Ginkgolic acid is a SUMOylation E1-E2-complex inhibitor that potently stimulates autophagy. Combining the high calcium concentrations indicative of PD and SUMOylation inhibition, TFEB would be free for nuclear translocation and promotion of lysosomal biogenesis.

As with late endosomes, autophagosomes incubate their cargo under acidic conditions to partially denature pH-sensitive proteins [104,167]. Acidic environments are maintained through ATP-dependent transmembrane proton pumps, which places strain on already dysfunctional mitochondria [204,205]. Moreover, the depletion of cellular ATP concentrations would further enhance AMPK, ultimately causing a positive feedback cycle of failing autophagosome development. Indeed, ferritin is an exceptionally durable and robust protein complex able to withstand a 2–10 pH range [121,206]. Ferritin accumulation in autophagosomes may therefore be acutely stable under conditions where α-synuclein inhibits lysosomal fusion events (Figure 6 and Figure 7). If the sole molecular mechanism that releases iron from ferritin (ferritinophagy) is inhibited, then a typical response would be to scavenge iron from external sources (i.e., upregulating expression of iron importing proteins). Excess intracellular iron will then be stored in ferritin thereby again becoming unavailable for cellular use. Moreover, progressive uptake of iron would lead to a “snowballing” accumulation of iron [207,208]. Autophagosomes chronically overloaded with ferritin may suffer from internal redox damage from ferritin denaturation, particularly given the acidic environment [209,210,211]. The catalytic reduction of Fe^3+^ will potentiate Fenton-Haber-Weiss chemistry and instigate lipid peroxidation. The presence of H_2_O_2_ within ferritin cores may further contribute to oxidation and membrane damage [121]. Membrane damage could become so severe that autophagosomes eventually rupture, expelling their contents into the cytosolic space. Since both α-synuclein [77] and β-amyloid [18] have been associated with the reduction of Fe^3+^, either aggregate may further contribute to redox-dependent damage and cell death.

Multiple mechanisms contribute to the cell-to-cell prion-like spread of α-synuclein [212,213,214]. Peripheral inoculation of neurons with pre-formed fibrils show a clear path of retroactive degeneration and increased presence of pSer129 α-synuclein [215]. Mitochondria and lysosomes can also become trafficked between proximal cells via tunnelling nanotubes and mediate intercellular α-synuclein transmission [130,216]. Neurons can secrete α-synuclein aggregates to the local extracellular environment, and the protease-resistance of α-synuclein aggregates enables translocation to distal sites after uptake by migrating microglia [9,217,218,219]. The failure of autophagosomes to degrade α-synuclein aggregates may result in trafficking to multivesicular bodies and secretion of α-synuclein in exosomes that can then mediate uptake by neighbouring cells [219,220,221]. These pathways may also contribute to the high ferritin content observed in glial cells in PD [222,223,224]. The iron content in the CSF of PD patients shows little difference to control samples [225], suggesting that it is the intrinsic handling of ferritin that leads to intercellular iron accumulation. Therefore, α-synuclein orchestrates an environment that enhances secretory pathways and prevents autophagic cargo degradation. This may lead to the mediation of cell-to-cell transmission of both α-synuclein and ferritin.

## 9. Therapeutic Targets for Ferritinophagy

An essential aim for therapeutics is to restore vesicular trafficking. In retinal pigmented epithelial cells, the overexpression of Rab1a rescued ferritinophagy following α-synuclein aggregation. These findings imply that rescuing Rab proteins and restoring vesicular trafficking may be enticing therapeutic avenues. Previous studies by Zhuang et al., (2020) described that pharmacological enhancement of transcription factor EB (TFEB) rescued SH-SY5Y cells from 6-OHDA toxicity [226]. Specifically, treatments of Torin1 and curcumin C1 analogue effectively enhanced TFEB translocation across the nuclear pore and thus, enhanced the expression of more than 500 genes associated with lysosomal biogenesis, autophagy, and vesicular trafficking [227]. Like AMPK and ULK1, TFEB is negatively regulated by mTOR serine/threonine kinase [228].

TFEB phosphorylation permits the binding of 14-3-3 binding proteins, ultimately inhibiting translocation across the nuclear pore (Figure 7). Calcineurin-dependent phosphate cleavage occurs in calcium-enriched environments, implicating lysosomal stress and calcium-exchange pumps [229]. In PD, intracellular calcium concentrations are elevated, yet calcineurin activity is not sufficient to promote TFEB translocation [37,75,230,231]. However, calcineurin has not been a successful therapeutic target [232]. Small-ubiquitin-like modifier 1 (SUMO1) has been shown to modify L347 on TFEB and inhibit translocation [233]. This block requires sentrin-specific protease (SENP) for SUMO cleavage and effective TFEB translocation. It may be that SENP3 activity is insufficient to relieve the inhibition of TFEB translocation as a homeostatic mechanism [234,235]. Recent work has investigated the SUMOylation inhibitor, ginkgolic acid (GA) [205,236]. In cell culture models, GA can restore autophagy and facilitate the clearance of cytoplasmic α-synuclein aggregates [237]. Moreover, GA has been demonstrated to have anti-inflammatory properties by inhibiting cyclooxygenase-1 (COX1, also known as prostaglandin G/H synthase 1) and prostaglandin synthesis [238,239,240]. However, higher concentrations and prolonged incubation with GA can be cytotoxic [241]. Studies have recently focused on producing safer and more effective SUMOylation inhibitors for potential therapeutic use [242].

While TFEB modulation may be a significant factor involved in GA-mediated lysosomal biogenesis, the dynamic nature of the different autophagic pathways, paired with the multitude of modulators, has presented other possibilities. For example, various studies have established evidence that indicates change in Hsp90, a CMA co-chaperone, in a manner that concurrently promotes macroautophagy [243,244,245]. Hsp90 facilitates CMA via stabilizing LAMP-2A, while pharmacological inhibition of Hsp90 mitigates CMA [137]. GA-dependent inhibition of SUMOylation attenuates Hsp90-SUMO conjugates and, thus, Hsp90 activity by reducing the interaction with the co-chaperone, Aha1.

Investigations utilizing confocal immunofluorescent microscopy and Western blotting showed that while Hsp90 expression decreased after GA treatments, LC3-II is significantly upregulated [237]. This ideology supports the proposed inverse regulation between CMA and macroautophagy [246]. However, these studies suggest a direct shift from CMA to macroautophagy may result from GA treatment, mediated by Hsp90 inhibition.

## 10. Conclusions

The present article reviewed the toxicity and molecular mechanisms underlying the inhibition of vesicle trafficking in PD, and the role of α-synuclein have been reviewed in the present article. Elevated brain iron has long been recognized as a consistent feature of PD, yet no current therapy targets the molecular mechanisms underlying iron accumulation. Ferritinophagy is significantly inhibited in the presence of α-synuclein and considering this, agents such as GA that have shown promise at restoring aggrephagy may also potentially be able to restore ferritinophagy. Further studies will be required to understand the complex interactome network regulating iron homeostasis. However, uncovering how this is disturbed in PD at the molecular level may reveal future therapeutic targets.

## Figures and Tables

**Table 1:** LIR Motif in known LC3 Targets Compared to NCOA4 and Potential LIR Domains.

Protein	Residue Location (Residue Number)	Flanking Residues											Motif			
**Y-type**																
**FUND1**	18–21	S	D	D	D	S	Y-E-V-L	D	L	T	E	Y	Y	E	V	L
**CRY1**	273–276	K	L	T	D	L	Y-K-K-V	K	K	N	S	S	Y	K	K	V
**MAVS**	9–12	A	E	D	K	T	Y-K-Y-I	C	R	N	F	S	Y	K	Y	I
**MAPK15**	340–343	Y	R	S	R	V	Y-Q-M-I	L	E	C	G	G	Y	Q	M	I
**NCOA4**	71–74	R	E	V	W	L	Y-E-Q-V	D	L	I	T	Q	Y	E	Q	V
**NCOA4**	428–431	E	K	E	A	L	Y-K-W-L	L	K	K	E	G	Y	K	W	L
**F-type**																
**ULK1**	367–370	C	D	T	D	D	F-V-M-V	P	A	Q	F	P	F	V	M	V
**WDFY3**	3346–3349	D	E	K	D	G	F-I-F-V	N	Y	S	E	G	F	I	F	V
**ATG2A**	1362–1365	L	D	S	D	E	F-C-I-L	D	A	P	G	L	F	C	I	L
**ATG13**	444–447	N	T	H	D	D	F-V-M-I	D	F	K	P	A	F	V	M	I
**BECL1**	97–100	E	S	A	N	S	F-T-L-I	I	G	E	A	S	F	T	L	I
**DISC1**	210–213	A	F	T	S	S	F-S-F-I	R	L	S	L	G	F	S	F	I
**ATX3**	74–77	M	D	D	S	G	F-F-S-I	Q	V	I	S	N	F	F	S	I
**NCOA4**	99–102	S	L	L	G	Q	F-N-C-L	T	H	Q	L	E	F	N	C	L
**NCOA4**	154–157	Q	T	I	T	T	F-G-S-L	K	T	I	Q	I	F	G	S	L
**NCOA4**	328–331	E	T	S	E	K	F-K-L-L	F	Q	S	Y	N	F	K	L	L
**NCOA4**	486–489	R	I	A	D	S	F-Q-V-I	K	N	S	P	L	F	Q	V	I
**W-type**																
**NEDD4**	685–688	E	S	S	E	N	W-E-I-I	R	E	D	E	A	W	E	I	I
**p62**	338–341	G	G	D	D	D	W-T-H-L	S	S	K	E	V	W	T	H	L
**Htt**	3035–3038	S	M	V	R	D	W-V-M-L	S	L	S	N	F	W	V	M	L
Confirmed Motif		Positively Residues						Polar Residues								
Speculated Motif		Negatively Charged						Non-Polar Residues								

Comparison of LC3-interacting proteins that utilize a classical LIR motif (green) with the ferritinophagy-specific adaptor protein NCOA4 (yellow). LIRs are grouped depending upon the first residue (Y, F, or W). The NCOA4 region 71–74 (YEQV) and 428–431 (YKWL) has high homology to the canonical LIR Y-type motifs of (Y/F/W)-x-x-(L/I/V), including the flanking acidic residue. Additionally, the residues 99–102 (FNCL), 154–157 (FGSL), 328–331 (FKLL), and 486–489 (FQVI) area also homologous to the canonical LIR F-type motif. *C*-terminal regions 99–102 and 154–157 do not contain flanking acidic residues but upon cross-referencing, it was observed that neither Beclin1 nor DISC1 contained flanking acidic resides. *N*-terminal regions 328–331 and 486–489 do contain a flanking residue. In all, there are six independent regions that conform to the LIR stipulations. Mutational experiments are required to determine the functionality of the potential motif sequences. Green = confirmed LIR motif sequences, yellow = possible NCOA4 LIR motif regions based upon established criterion, pink = negatively charged residues, orange = positively charged residues, cyan = polarized residues, blue = non-polar residues. NCOA4: nuclear coactivator 4. Y-type motif positive, FUND1: FUN14 domain-containing protein 1, CRY1: Cryptochrome-1, MAVS: Mitochondrial antiviral-signaling protein, MAPK15: Mitogen-activated protein kinase 15, ULK1: Unc-51 like autophagy activating kinase 1, WDFY3: WD Repeat And FYVE Domain Containing 3, ATG2A: Autophagy protein 2A, ATG13: Autophagy protein 13, BECL1: Beclin1, DISC1: Disrupted in schizophrenia 1, ATX3: Ataxin 3, NEDD4: E3-ubiquitin protein ligase neuronal precursor cell-expressed developmentally downregulated 4, p62: Sequestosome 1, Htt: Huntingtin.

## Abbreviations

AAAD	Aromatic amino acid decarboxylase
AD	Alzheimer’s disease
AKT1	RAC-alpha serine/threonine-protein kinase
ALS	Amyotrophic lateral sclerosis
AMBRA1	Autophagy and beclin-1 regulator 1
AMP	Adenosine monophosphate
AMPK	AMP protein kinase
AP1/2	Adaptor protein ½
APP	Amyloid precursor protein
Arp2/3	Actin-related proteins 2/3
ATG9	Autophagy-related protein 9
ATG13	Autophagy-related protein 13
ATG101	Autophagy-related protein 101
BARA	β-α Repeated autophagy
BCL-2	Apoptosis regulator B-cell lymphoma 2 Bcl-2
BCL-XL	B-cell lymphoma-extra large
CALB1	Calbindin-D28K
Ca_v_1.2	Voltage-dependent L-type calcium channel subunit α-1C
Ca_v_1.3	Calcium channel, voltage-dependent, L-type, α-1D subunit
CCD	Coiled-coil domain
CI-MPR	Cation-independent-mannose-6-phosphate receptor
CMA	Chaperone-mediated autophagy
COX-1	Cyclooxygenase-1
CSF	Cerebral spinal fluid
CSPα	Cysteine-string protein-α
DAT	Dopamine transporter
DLB	Dementia with Lewy bodies
DMT1	Divalent metal transporter 1
ECD	Evolutionarily conserved domain
ER	Endoplasmic reticulum
FAK	Focal adhesion kinase
Fe-S	Iron-sulfur cluster
FIP200	FAK family kinase-interacting protein of 200 kDa
FPN1	Ferroportin
Ft	Ferritin
FtH1	Ferritin heavy chain
FtL	Ferritin light chain
FTLD	Frontotemporal lobar dementia
FtMt	Mitochondrial ferritin
GA	Ginkgolic acid
GABA	γ-Aminobutyric acid
GABARAP	γ-Aminobutyric acid receptor-associated protein
GABARAPL1	γ-Aminobutyric acid receptor-associated protein-like 1
GABARAPL2	γ-Aminobutyric acid receptor-associated protein-like 2
GABRR3	γ-Aminobutyric acid receptor subunit rho-3
HD	Huntington’s disease
HERC2	E3 ubiquitin protein ligase 2
HOPS	Homotypic fusion and vacuole protein sorting
HP1	Hydrophobic pocket 1
HRB	HIV-1 Rev-binding-protein
Hsc70	Heat shock cognate 70 kDa
Hsp	Heat shock protein
Htt	Huntingtin
IRE	Iron-responsive element
IRP1	Iron regulatory protein 1
IMM	Inner-mitochondrial membrane
KO	Knockout
LAMP2a	Lysosomal-associated membrane protein 2a
LB	Lewy body
LC3	Microtubule-associated proteins 1A/1B light chain 3
LIP	Labile iron pool
LIR	LC3 interacting region
LN	Lewy neurites
MPTP	1-methyl-4-phenyl-1,2,3,6-tetrahydropyridine
MRI	Magnetic resonance imaging
MSA	Multiple system atrophy
mTOR	Mammalian target of rapamycin
mTORC1	mTOR complex 1
Munc-13	Mammalian uncoordinated 13
Munc-18	Mammalian uncoordinated 18
MVB	Multi-vesicular body
MWW	Weighted-average molecular weight
NAC	Non-amyloid component
NBIA5	Neurodegeneration with brain iron accumulation 5
NCOA4	Nuclear co-activator 4
NLRP3	NOD-like receptor protein 3
NOD	Nucleotide binding domain
Nrf2	Nuclear factor erythroid 2–related factor 2
6-OHDA	6-Hydroxydopamine
PAWR	PRKC-apoptosis-WT1-regulator
PCBP2	Poly-r(C)-binding protein 2
PD	Parkinson’s disease
PE	Phosphatidylethanolamine
PI3KC3	Phosphatidylinositol 3-kinase catalytic subunit type 3
PI3P	Phosphatidylinositol 3-phosphate
PNN	Pedunculopontine nucleus
Rab1a	RAS-related protein in brain 1a
Rab7	RAS-related protein Rab-7a
RB1CC1	RB1-inducible coiled-coil protein 1
RPE	Retinal pigmented epithelial cells
SENP	Sentrin-specific protease
SGT	Small glutamate-rich tetratricopeptide repeat-containing protein
SMV	Small single membrane vesicles
SNAP25	Synaptosomal-associated protein of 25 kDa
SNARE	Soluble N-ethylmaleimide sensitive attachment factor receptor
SNpc	Substantia nigra pars compacta
SNpr	Substantia nigra pars reticulata
SQSTM1	Sequestosome-1 (p62)
STEAP1	Six transmembrane epithelial antigen of the prostate-1
STX17	Syntaxin-17
SUMO1	Small-ubiquitin-like modifier 1
SYNGAP	Synaptic Ras-like GTPase-activating protein 1
TBC1D5	TBC1 domain family member 5
TFEB	Transcription factor EB
TFR1	Transferrin receptor 1
TFR2	Transferrin receptor 2
TH	Tyrosine hydroxylase
TGN	*trans*-Golgi network
TLR4	Toll-like receptor 4
TOM20	Translocase of outer membrane of 20 kDa
TOMM20	Mitochondrial import receptor subunit TOM20 homolog
t-SNARE	Target SNARE
UBA	Ubiquitin-associated
ULK1	Unc-51 like autophagy activating kinase
Unc-51	Serine/threonine-protein kinase unc-51
UPS	Ubiquitin/proteasome system
UVRAG	UV radiation resistance-associated gene protein
VAMP2	Vesicle-associated membrane protein 2 (also known as synaptobrevin)
VGCC	Voltage-gated calcium channel
VMAT2	Vesicular-monoamine transporter
VPS26	Vacuolar protein sorting-associated protein 26A
VPS29	Vacuolar protein sorting-associated protein 29
VPS35	VPS35 endosomal protein-sorting factor-like
v-SNARE	Vesicular SNARE
WASH	Wiskott–Aldrich syndrome protein and SCAR homologue
WIPI2	WD repeat domain phosphoinositide-interacting protein 2

## References

[1] Parkinson J. (2002). An essay on the shaking palsy. J. Neuropsychiatry Clin. Neurosci..

[2] Watanabe I., Vachal E., Tomita T. (1977). Dense core vesicles around the Lewy body in incidental Parkinson’s disease: An electron microscopic study. Acta Neuropathol..

[3] Spillantini M.G., Schmidt M.L., Lee V.M., Trojanowski J.Q., Jakes R., Goedert M. (1997). Alpha-synuclein in Lewy bodies. Nature.

[4] Mochizuki H., Choong C.J., Baba K. (2020). Parkinson’s disease and iron. J. Neural Transm.

[5] Singh P.K., Muqit M.M.K. (2020). Parkinson’s: A Disease of Aberrant Vesicle Trafficking. Annu. Rev. Cell Dev. Biol..

[6] Abeyawardhane D.L., Lucas H.R. (2019). Iron Redox Chemistry and Implications in the Parkinson’s Disease Brain. Oxid. Med. Cell Longev..

[7] Dexter D.T., Carayon A., Javoy-Agid F., Agid Y., Wells F.R., Daniel S.E., Lees A.J., Jenner P., Marsden C.D. (1991). Alterations in the levels of iron, ferritin and other trace metals in Parkinson’s disease and other neurodegenerative diseases affecting the basal ganglia. Brain.

[8] Zecca L., Shima T., Stroppolo A., Goj C., Battiston G.A., Gerbasi R., Sarna T., Swartz H.M. (1996). Interaction of neuromelanin and iron in substantia nigra and other areas of human brain. Neuroscience.

[9] Zhang W., Wang T., Pei Z., Miller D.S., Wu X., Block M.L., Wilson B., Zhang W., Zhou Y., Hong J.S. (2005). Aggregated alpha-synuclein activates microglia: A process leading to disease progression in Parkinson’s disease. FASEB J..

[10] Kaji S., Maki T., Ishimoto T., Yamakado H., Takahashi R. (2020). Insights into the pathogenesis of multiple system atrophy: Focus on glial cytoplasmic inclusions. Transl. Neurodegener..

[11] Calo L., Wegrzynowicz M., Santivanez-Perez J., Grazia Spillantini M. (2016). Synaptic failure and alpha-synuclein. Mov. Disord..

[12] Di Marco Vieira B., Radford R.A.W., Hayashi J., Eaton E.D., Greenaway B., Jambas M., Petcu E.B., Chung R.S., Pountney D.L. (2020). Extracellular Alpha-Synuclein Promotes a Neuroinhibitory Secretory Phenotype in Astrocytes. Life.

[13] Moons R., Konijnenberg A., Mensch C., Van Elzen R., Johannessen C., Maudsley S., Lambeir A.M., Sobott F. (2020). Metal ions shape alpha-synuclein. Sci. Rep..

[14] Han J., Day J.R., Connor J.R., Beard J.L. (2002). H and L ferritin subunit mRNA expression differs in brains of control and iron-deficient rats. J. Nutr..

[15] Rouault T.A., Zhang D.L., Jeong S.Y. (2009). Brain iron homeostasis, the choroid plexus, and localization of iron transport proteins. Metab. Brain Dis..

[16] Meyron-Holtz E.G., Cohen L.A., Fahoum L., Haimovich Y., Lifshitz L., Magid-Gold I., Stuemler T., Truman-Rosentsvit M. (2014). Ferritin polarization and iron transport across monolayer epithelial barriers in mammals. Front. Pharmacol..

[17] Truman-Rosentsvit M., Berenbaum D., Spektor L., Cohen L.A., Belizowsky-Moshe S., Lifshitz L., Ma J., Li W., Kesselman E., Abutbul-Ionita I. (2018). Ferritin is secreted via 2 distinct nonclassical vesicular pathways. Blood.

[18] Everett J., Brooks J., Lermyte F., O’Connor P.B., Sadler P.J., Dobson J., Collingwood J.F., Telling N.D. (2020). Iron stored in ferritin is chemically reduced in the presence of aggregating Abeta(1-42). Sci. Rep..

[19] Santana-Codina N., Mancias J.D. (2018). The Role of NCOA4-Mediated Ferritinophagy in Health and Disease. Pharmaceuticals.

[20] Tang M., Chen Z., Wu D., Chen L. (2018). Ferritinophagy/ferroptosis: Iron-related newcomers in human diseases. J. Cell Physiol..

[21] Quiles Del Rey M., Mancias J.D. (2019). NCOA4-Mediated Ferritinophagy: A Potential Link to Neurodegeneration. Front. Neurosci..

[22] Hodge G.K., Butcher L.L. (1980). Pars compacta of the substantia nigra modulates motor activity but is not involved importantly in regulating food and water intake. Naunyn. Schmiedebergs Arch. Pharmacol..

[23] Fabbri M., Reimao S., Carvalho M., Nunes R.G., Abreu D., Guedes L.C., Bouca R., Lobo P.P., Godinho C., Coelho M. (2017). Substantia Nigra Neuromelanin as an Imaging Biomarker of Disease Progression in Parkinson’s Disease. J. Parkinsons Dis..

[24] Liang C.L., Sinton C.M., Sonsalla P.K., German D.C. (1996). Midbrain dopaminergic neurons in the mouse that contain calbindin-D28k exhibit reduced vulnerability to MPTP-induced neurodegeneration. Neurodegeneration.

[25] de Berker A.O., Rutledge R.B. (2014). A role for the human substantia nigra in reinforcement learning. J. Neurosci..

[26] Galtieri D.J., Estep C.M., Wokosin D.L., Traynelis S., Surmeier D.J. (2017). Pedunculopontine glutamatergic neurons control spike patterning in substantia nigra dopaminergic neurons. Elife.

[27] Sonne J., Reddy V., Beato M.R. (2021). Neuroanatomy, Substantia Nigra. StatPearls.

[28] Brichta L., Greengard P. (2014). Molecular determinants of selective dopaminergic vulnerability in Parkinson’s disease: An update. Front. Neuroanat..

[29] Lu B., Palacino J. (2013). A novel human embryonic stem cell-derived Huntington’s disease neuronal model exhibits mutant huntingtin (mHTT) aggregates and soluble mHTT-dependent neurodegeneration. FASEB J..

[30] Saudou F., Humbert S. (2016). The Biology of Huntingtin. Neuron.

[31] Melland H., Carr E.M., Gordon S.L. (2021). Disorders of synaptic vesicle fusion machinery. J. Neurochem..

[32] Bradbury A., Bagel J., Sampson M., Farhat N., Ding W., Swain G., Prociuk M., O’Donnell P., Drobatz K., Gurda B. (2016). Cerebrospinal Fluid Calbindin D Concentration as a Biomarker of Cerebellar Disease Progression in Niemann-Pick Type C1 Disease. J. Pharmacol. Exp. Ther..

[33] Wolff N.A., Ghio A.J., Garrick L.M., Garrick M.D., Zhao L., Fenton R.A., Thevenod F. (2014). Evidence for mitochondrial localization of divalent metal transporter 1 (DMT1). FASEB J..

[34] Du X., Xu H., Shi L., Jiang Z., Song N., Jiang H., Xie J. (2016). Activation of ATP-sensitive potassium channels enhances DMT1-mediated iron uptake in SK-N-SH cells in vitro. Sci. Rep..

[35] Bazelon M., Fenichel G.M., Randall J. (1967). Studies on neuromelanin. I. A melanin system in the human adult brainstem. Neurology.

[36] Carballo-Carbajal I., Laguna A., Romero-Gimenez J., Cuadros T., Bove J., Martinez-Vicente M., Parent A., Gonzalez-Sepulveda M., Penuelas N., Torra A. (2019). Brain tyrosinase overexpression implicates age-dependent neuromelanin production in Parkinson’s disease pathogenesis. Nat. Commun..

[37] Lautenschlager J., Stephens A.D., Fusco G., Strohl F., Curry N., Zacharopoulou M., Michel C.H., Laine R., Nespovitaya N., Fantham M. (2018). C-terminal calcium binding of alpha-synuclein modulates synaptic vesicle interaction. Nat. Commun..

[38] Burre J., Sharma M., Tsetsenis T., Buchman V., Etherton M.R., Sudhof T.C. (2010). Alpha-synuclein promotes SNARE-complex assembly in vivo and in vitro. Science.

[39] Trexler A.J., Rhoades E. (2012). N-Terminal acetylation is critical for forming alpha-helical oligomer of alpha-synuclein. Protein Sci..

[40] Deng S., Pan B., Gottlieb L., Petersson E.J., Marmorstein R. (2020). Molecular basis for N-terminal alpha-synuclein acetylation by human NatB. Elife.

[41] Bartels T., Ahlstrom L.S., Leftin A., Kamp F., Haass C., Brown M.F., Beyer K. (2010). The N-terminus of the intrinsically disordered protein alpha-synuclein triggers membrane binding and helix folding. Biophys. J..

[42] Chen R.H.C., Wislet-Gendebien S., Samuel F., Visanji N.P., Zhang G., Marsilio D., Langman T., Fraser P.E., Tandon A. (2013). alpha-Synuclein membrane association is regulated by the Rab3a recycling machinery and presynaptic activity. J. Biol. Chem..

[43] Siddiqui I.J., Pervaiz N., Abbasi A.A. (2016). The Parkinson Disease gene SNCA: Evolutionary and structural insights with pathological implication. Sci. Rep..

[44] Appel-Cresswell S., Vilarino-Guell C., Encarnacion M., Sherman H., Yu I., Shah B., Weir D., Thompson C., Szu-Tu C., Trinh J. (2013). Alpha-synuclein p.H50Q, a novel pathogenic mutation for Parkinson’s disease. Mov. Disord..

[45] Lesage S., Anheim M., Letournel F., Bousset L., Honore A., Rozas N., Pieri L., Madiona K., Durr A., Melki R. (2013). G51D alpha-synuclein mutation causes a novel parkinsonian-pyramidal syndrome. Ann. Neurol..

[46] Kruger R., Kuhn W., Muller T., Woitalla D., Graeber M., Kosel S., Przuntek H., Epplen J.T., Schols L., Riess O. (1998). Ala30Pro mutation in the gene encoding alpha-synuclein in Parkinson’s disease. Nat. Genet..

[47] Polymeropoulos M.H., Lavedan C., Leroy E., Ide S.E., Dehejia A., Dutra A., Pike B., Root H., Rubenstein J., Boyer R. (1997). Mutation in the alpha-synuclein gene identified in families with Parkinson’s disease. Science.

[48] Zarranz J.J., Alegre J., Gomez-Esteban J.C., Lezcano E., Ros R., Ampuero I., Vidal L., Hoenicka J., Rodriguez O., Atares B. (2004). The new mutation, E46K, of alpha-synuclein causes Parkinson and Lewy body dementia. Ann. Neurol..

[49] Hong W., Lev S. (2014). Tethering the assembly of SNARE complexes. Trends Cell Biol..

[50] Hawk B.J.D., Khounlo R., Shin Y.K. (2019). Alpha-Synuclein Continues to Enhance SNARE-Dependent Vesicle Docking at Exorbitant Concentrations. Front. Neurosci..

[51] Sun J., Wang L., Bao H., Premi S., Das U., Chapman E.R., Roy S. (2019). Functional cooperation of alpha-synuclein and VAMP2 in synaptic vesicle recycling. Proc. Natl. Acad. Sci. USA.

[52] Schoch S., Deak F., Konigstorfer A., Mozhayeva M., Sara Y., Sudhof T.C., Kavalali E.T. (2001). SNARE function analyzed in synaptobrevin/VAMP knockout mice. Science.

[53] Koo S.J., Markovic S., Puchkov D., Mahrenholz C.C., Beceren-Braun F., Maritzen T., Dernedde J., Volkmer R., Oschkinat H., Haucke V. (2011). SNARE motif-mediated sorting of synaptobrevin by the endocytic adaptors clathrin assembly lymphoid myeloid leukemia (CALM) and AP180 at synapses. Proc. Natl. Acad. Sci. USA.

[54] Burre J., Sharma M., Sudhof T.C. (2014). alpha-Synuclein assembles into higher-order multimers upon membrane binding to promote SNARE complex formation. Proc. Natl. Acad. Sci. USA.

[55] Burgoyne R.D., Morgan A. (2015). Cysteine string protein (CSP) and its role in preventing neurodegeneration. Semin. Cell Dev. Biol..

[56] Sharma M., Burre J., Sudhof T.C. (2011). CSPalpha promotes SNARE-complex assembly by chaperoning SNAP-25 during synaptic activity. Nat. Cell Biol..

[57] Chandra S., Gallardo G., Fernandez-Chacon R., Schluter O.M., Sudhof T.C. (2005). Alpha-synuclein cooperates with CSPalpha in preventing neurodegeneration. Cell.

[58] Sharma M., Burre J., Bronk P., Zhang Y., Xu W., Sudhof T.C. (2012). CSPalpha knockout causes neurodegeneration by impairing SNAP-25 function. EMBO J..

[59] Bandyopadhyay U., Kaushik S., Varticovski L., Cuervo A.M. (2008). The chaperone-mediated autophagy receptor organizes in dynamic protein complexes at the lysosomal membrane. Mol. Cell Biol..

[60] Zhou P., Pang Z.P., Yang X., Zhang Y., Rosenmund C., Bacaj T., Sudhof T.C. (2013). Syntaxin-1 N-peptide and Habc-domain perform distinct essential functions in synaptic vesicle fusion. EMBO J..

[61] Wang S., Li Y., Gong J., Ye S., Yang X., Zhang R., Ma C. (2019). Munc18 and Munc13 serve as a functional template to orchestrate neuronal SNARE complex assembly. Nat. Commun..

[62] Shao K., Li F., Yang Y., Wang N., Gao X.D., Nakanishi H. (2020). Characteristics of SNARE proteins are defined by distinctive properties of SNARE motifs. Biochim. Biophys. Acta Gen. Subj..

[63] Chai Y.J., Sierecki E., Tomatis V.M., Gormal R.S., Giles N., Morrow I.C., Xia D., Gotz J., Parton R.G., Collins B.M. (2016). Munc18-1 is a molecular chaperone for alpha-synuclein, controlling its self-replicating aggregation. J. Cell Biol..

[64] Rao S.K., Huynh C., Proux-Gillardeaux V., Galli T., Andrews N.W. (2004). Identification of SNAREs involved in synaptotagmin VII-regulated lysosomal exocytosis. J. Biol. Chem..

[65] Lee H.K., Yang Y., Su Z., Hyeon C., Lee T.S., Lee H.W., Kweon D.H., Shin Y.K., Yoon T.Y. (2010). Dynamic Ca2+-dependent stimulation of vesicle fusion by membrane-anchored synaptotagmin 1. Science.

[66] Zhou Q., Zhou P., Wang A.L., Wu D., Zhao M., Sudhof T.C., Brunger A.T. (2017). The primed SNARE-complexin-synaptotagmin complex for neuronal exocytosis. Nature.

[67] Abeliovich A., Schmitz Y., Farinas I., Choi-Lundberg D., Ho W.H., Castillo P.E., Shinsky N., Verdugo J.M., Armanini M., Ryan A. (2000). Mice lacking alpha-synuclein display functional deficits in the nigrostriatal dopamine system. Neuron.

[68] Greten-Harrison B., Polydoro M., Morimoto-Tomita M., Diao L., Williams A.M., Nie E.H., Makani S., Tian N., Castillo P.E., Buchman V.L. (2010). alphabetagamma-Synuclein triple knockout mice reveal age-dependent neuronal dysfunction. Proc. Natl. Acad. Sci. USA.

[69] Butler B., Saha K., Rana T., Becker J.P., Sambo D., Davari P., Goodwin J.S., Khoshbouei H. (2015). Dopamine Transporter Activity Is Modulated by alpha-Synuclein. J. Biol. Chem..

[70] Ishibashi K., Oda K., Ishiwata K., Ishii K. (2014). Comparison of dopamine transporter decline in a patient with Parkinson’s disease and normal aging effect. J. Neurol. Sci..

[71] Egana L.A., Cuevas R.A., Baust T.B., Parra L.A., Leak R.K., Hochendoner S., Pena K., Quiroz M., Hong W.C., Dorostkar M.M. (2009). Physical and functional interaction between the dopamine transporter and the synaptic vesicle protein synaptogyrin-.-3. J. Neurosci..

[72] Cooper A.A. (2006). Synuclein Blocks ER-Golgi Traffic and Rab1 Rescues Neuron Loss in Parkinson’s Models. Science.

[73] Baksi S., Singh N. (2017). alpha-Synuclein impairs ferritinophagy in the retinal pigment epithelium: Implications for retinal iron dyshomeostasis in Parkinson’s disease. Sci. Rep..

[74] Leandrou E., Emmanouilidou E., Vekrellis K. (2019). Voltage-Gated Calcium Channels and alpha-Synuclein: Implications in Parkinson’s Disease. Front. Mol. Neurosci..

[75] Llinas R., Sugimori M., Silver R.B. (1992). Microdomains of high calcium concentration in a presynaptic terminal. Science.

[76] Okita Y., Rcom-H’cheo-Gauthier A.N., Goulding M., Chung R.S., Faller P., Pountney D.L. (2017). Metallothionein, copper and alpha-Synuclein in alpha-synucleinopathies. Front. Neurosci..

[77] Davies P., Moualla D., Brown D.R. (2011). Alpha-synuclein is a cellular ferrireductase. PLoS ONE.

[78] Binolfi A., Rasia R.M., Bertoncini C.W., Ceolin M., Zweckstetter M., Griesinger C., Jovin T.M., Fernandez C.O. (2006). Interaction of alpha-synuclein with divalent metal ions reveals key differences: A link between structure, binding specificity and fibrillation enhancement. J. Am. Chem. Soc..

[79] Chen B., Wen X., Jiang H., Wang J., Song N., Xie J. (2019). Interactions between iron and alpha-synuclein pathology in Parkinson’s disease. Free Radic. Biol. Med..

[80] Ureshino R.P., Erustes A.G., Bassani T.B., Wachilewski P., Guarache G.C., Nascimento A.C., Costa A.J., Smaili S.S., Pereira G. (2019). The Interplay between Ca(2+) Signaling Pathways and Neurodegeneration. Int. J. Mol. Sci..

[81] Serpell L.C., Berriman J., Jakes R., Goedert M., Crowther R.A. (2000). Fiber diffraction of synthetic alpha-synuclein filaments shows amyloid-like cross-beta conformation. Proc. Natl. Acad. Sci. USA.

[82] Pountney D.L., Voelcker N.H., Gai W.P. (2005). Annular alpha-synuclein oligomers are potentially toxic agents in alpha-synucleinopathy. Hypothesis. Neurotox. Res..

[83] Guiney S.J., Adlard P.A., Lei P., Mawal C.H., Bush A.I., Finkelstein D.I., Ayton S. (2020). Fibrillar alpha-synuclein toxicity depends on functional lysosomes. J. Biol. Chem..

[84] Hindeya Gebreyesus H., Gebrehiwot Gebremichael T. (2020). The Potential Role of Astrocytes in Parkinson’s Disease (PD). Med. Sci..

[85] Friedlich A.L., Tanzi R.E., Rogers J.T. (2007). The 5’-untranslated region of Parkinson’s disease alpha-synuclein messengerRNA contains a predicted iron responsive element. Mol. Psychiatry.

[86] Ma L., Gholam Azad M., Dharmasivam M., Richardson V., Quinn R.J., Feng Y., Pountney D.L., Tonissen K.F., Mellick G.D., Yanatori I. (2021). Parkinson’s disease: Alterations in iron and redox biology as a key to unlock therapeutic strategies. Redox Biol..

[87] Hare D.J., Double K.L. (2016). Iron and dopamine: A toxic couple. Brain.

[88] Zucca F.A., Segura-Aguilar J., Ferrari E., Munoz P., Paris I., Sulzer D., Sarna T., Casella L., Zecca L. (2017). Interactions of iron, dopamine and neuromelanin pathways in brain aging and Parkinson’s disease. Prog. Neurobiol..

[89] Shima T., Sarna T., Swartz H.M., Stroppolo A., Gerbasi R., Zecca L. (1997). Binding of iron to neuromelanin of human substantia nigra and synthetic melanin: An electron paramagnetic resonance spectroscopy study. Free Radic. Biol. Med..

[90] Ren J.X., Sun X., Yan X.L., Guo Z.N., Yang Y. (2020). Ferroptosis in Neurological Diseases. Front. Cell Neurosci..

[91] Zecca L., Stroppolo A., Gatti A., Tampellini D., Toscani M., Gallorini M., Giaveri G., Arosio P., Santambrogio P., Fariello R.G. (2004). The role of iron and copper molecules in the neuronal vulnerability of locus coeruleus and substantia nigra during aging. Proc. Natl. Acad. Sci. USA.

[92] Sulzer D., Cassidy C., Horga G., Kang U.J., Fahn S., Casella L., Pezzoli G., Langley J., Hu X.P., Zucca F.A. (2018). Neuromelanin detection by magnetic resonance imaging (MRI) and its promise as a biomarker for Parkinson’s disease. NPJ Parkinsons Dis..

[93] Vila M., Laguna A., Carballo-Carbajal I. (2019). Intracellular crowding by age-dependent neuromelanin accumulation disrupts neuronal proteostasis and triggers Parkinson disease pathology. Autophagy.

[94] Bjorkoy G., Lamark T., Brech A., Outzen H., Perander M., Overvatn A., Stenmark H., Johansen T. (2005). p62/SQSTM1 forms protein aggregates degraded by autophagy and has a protective effect on huntingtin-induced cell death. J. Cell Biol..

[95] del Toro D., Alberch J., Lazaro-Dieguez F., Martin-Ibanez R., Xifro X., Egea G., Canals J.M. (2009). Mutant huntingtin impairs post-Golgi trafficking to lysosomes by delocalizing optineurin/Rab8 complex from the Golgi apparatus. Mol. Biol. Cell.

[96] Anekonda T.S., Quinn J.F. (2011). Calcium channel blocking as a therapeutic strategy for Alzheimer’s disease: The case for isradipine. Biochim. Biophys. Acta.

[97] Cho S.J., Yun S.M., Jo C., Lee D.H., Choi K.J., Song J.C., Park S.I., Kim Y.J., Koh Y.H. (2015). SUMO1 promotes Abeta production via the modulation of autophagy. Autophagy.

[98] Tadokoro K., Yamashita T., Shang J., Ohta Y., Nomura E., Morihara R., Omote Y., Takemoto M., Abe K. (2021). Switching the Proteolytic System from the Ubiquitin-Proteasome System to Autophagy in the Spinal Cord of an Amyotrophic Lateral Sclerosis Mouse Model. Neuroscience.

[99] Mayle K.M., Le A.M., Kamei D.T. (2012). The intracellular trafficking pathway of transferrin. Biochim. Biophys. Acta.

[100] Harding C., Heuser J., Stahl P. (1983). Receptor-mediated endocytosis of transferrin and recycling of the transferrin receptor in rat reticulocytes. J. Cell Biol..

[101] Wilson R.L., Wightman R.M. (1985). Systemic and Nigral Application of Amphetamine Both Cause an Increase in Extracellular Concentration of Ascorbate in the Caudate-Nucleus of the Rat. Brain Res..

[102] Schenk J.O., Miller E., Gaddis R., Adams R.N. (1982). Homeostatic control of ascorbate concentration in CNS extracellular fluid. Brain Res..

[103] Boag M.K., Ma L., Mellick G.D., Pountney D.L., Feng Y., Quinn R.J., Liew A.W.-C., Dharmasivam M., Azad M.G., Afroz R. (2021). Calcium channels and iron metabolism: A redox catastrophe in Parkinson’s disease and an innovative path to novel therapies?. Redox Biol..

[104] Birgisdottir A.B., Johansen T. (2020). Autophagy and endocytosis—interconnections and interdependencies. J. Cell Sci..

[105] Peng X., Yang L., Ma Y., Li X., Yang S., Li Y., Wu B., Tang S., Zhang F., Zhang B. (2021). IKKbeta activation promotes amphisome formation and extracellular vesicle secretion in tumor cells. Biochim. Biophys. Acta Mol. Cell Res..

[106] Hill S.E., Colon-Ramos D.A. (2020). The Journey of the Synaptic Autophagosome: A Cell Biological Perspective. Neuron.

[107] Ohgami R.S., Campagna D.R., McDonald A., Fleming M.D. (2006). The Steap proteins are metalloreductases. Blood.

[108] Al-Refaei M.A., Makki R.M., Ali H.M. (2020). Structure prediction of transferrin receptor protein 1 (TfR1) by homology modelling, docking, and molecular dynamics simulation studies. Heliyon.

[109] Salazar J., Mena N., Hunot S., Prigent A., Alvarez-Fischer D., Arredondo M., Duyckaerts C., Sazdovitch V., Zhao L., Garrick L.M. (2008). Divalent metal transporter 1 (DMT1) contributes to neurodegeneration in animal models of Parkinson’s disease. Proc. Natl. Acad. Sci. USA.

[110] Skjorringe T., Burkhart A., Johnsen K.B., Moos T. (2015). Divalent metal transporter 1 (DMT1) in the brain: Implications for a role in iron transport at the blood-brain barrier, and neuronal and glial pathology. Front. Mol. Neurosci..

[111] Bi M., Du X., Jiao Q., Liu Z., Jiang H. (2020). alpha-Synuclein Regulates Iron Homeostasis via Preventing Parkin-Mediated DMT1 Ubiquitylation in Parkinson’s Disease Models. ACS Chem. Neurosci..

[112] Tchernitchko D., Bourgeois M., Martin M.E., Beaumont C. (2002). Expression of the two mRNA isoforms of the iron transporter Nramp2/DMTI in mice and function of the iron responsive element. Biochem. J..

[113] Foot N.J., Dalton H.E., Shearwin-Whyatt L.M., Dorstyn L., Tan S.S., Yang B., Kumar S. (2008). Regulation of the divalent metal ion transporter DMT1 and iron homeostasis by a ubiquitin-dependent mechanism involving Ndfips and WWP2. Blood.

[114] You F., Sun H., Zhou X., Sun W., Liang S., Zhai Z., Jiang Z. (2009). PCBP2 mediates degradation of the adaptor MAVS via the HECT ubiquitin ligase AIP4. Nat. Immunol..

[115] Leidgens S., Bullough K.Z., Shi H., Li F., Shakoury-Elizeh M., Yabe T., Subramanian P., Hsu E., Natarajan N., Nandal A. (2013). Each member of the poly-r(C)-binding protein 1 (PCBP) family exhibits iron chaperone activity toward ferritin. J. Biol. Chem..

[116] Frey A.G., Nandal A., Park J.H., Smith P.M., Yabe T., Ryu M.S., Ghosh M.C., Lee J., Rouault T.A., Park M.H. (2014). Iron chaperones PCBP1 and PCBP2 mediate the metallation of the dinuclear iron enzyme deoxyhypusine hydroxylase. Proc. Natl. Acad. Sci. USA.

[117] Yanatori I., Richardson D.R., Toyokuni S., Kishi F. (2020). The new role of poly (rC)-binding proteins as iron transport chaperones: Proteins that could couple with inter-organelle interactions to safely traffic iron. Biochim. Biophys. Acta Gen. Subj..

[118] Yanatori I., Richardson D.R., Toyokuni S., Kishi F. (2017). The iron chaperone poly(rC)-binding protein 2 forms a metabolon with the heme oxygenase 1/cytochrome P450 reductase complex for heme catabolism and iron transfer. J. Biol. Chem..

[119] Bae D.H., Lane D.J.R., Siafakas A.R., Sutak R., Paluncic J., Huang M.L.H., Jansson P.J., Rahmanto Y.S., Richardson D.R. (2020). Acireductone dioxygenase 1 (ADI1) is regulated by cellular iron by a mechanism involving the iron chaperone, PCBP1, with PCBP2 acting as a potential co-chaperone. Biochim. Biophys. Acta Mol. Basis Dis..

[120] Harrison P.M. (1986). The structure and function of ferritin. Biochem. Educ..

[121] Melman A., Bou-Abdallah F. (2020). Iron mineralization and core dissociation in mammalian homopolymeric H-ferritin: Current understanding and future perspectives. Biochim. Biophys. Acta Gen. Subj..

[122] Arosio P., Elia L., Poli M. (2017). Ferritin, cellular iron storage and regulation. IUBMB Life.

[123] Campanella A., Rovelli E., Santambrogio P., Cozzi A., Taroni F., Levi S. (2009). Mitochondrial ferritin limits oxidative damage regulating mitochondrial iron availability: Hypothesis for a protective role in Friedreich ataxia. Hum. Mol. Genet..

[124] Guan H., Yang H., Yang M., Yanagisawa D., Bellier J.P., Mori M., Takahata S., Nonaka T., Zhao S., Tooyama I. (2017). Mitochondrial ferritin protects SH-SY5Y cells against H2O2-induced oxidative stress and modulates alpha-synuclein expression. Exp. Neurol..

[125] Yang Y., Fiskus W., Yong B., Atadja P., Takahashi Y., Pandita T.K., Wang H.G., Bhalla K.N. (2013). Acetylated hsp70 and KAP1-mediated Vps34 SUMOylation is required for autophagosome creation in autophagy. Proc. Natl. Acad. Sci. USA.

[126] Chiang S., Huang M.L.H., Park K.C., Richardson D.R. (2020). Antioxidant defense mechanisms and its dysfunctional regulation in the mitochondrial disease, Friedreich’s ataxia. Free Radic. Biol. Med..

[127] Perfeito R., Lazaro D.F., Outeiro T.F., Rego A.C. (2014). Linking alpha-synuclein phosphorylation to reactive oxygen species formation and mitochondrial dysfunction in SH-SY5Y cells. Mol. Cell Neurosci..

[128] Chinta S.J., Mallajosyula J.K., Rane A., Andersen J.K. (2010). Mitochondrial alpha-synuclein accumulation impairs complex I function in dopaminergic neurons and results in increased mitophagy in vivo. Neurosci. Lett..

[129] Valdinocci D., Simoes R.F., Kovarova J., Cunha-Oliveira T., Neuzil J., Pountney D.L. (2019). Intracellular and Intercellular Mitochondrial Dynamics in Parkinson’s Disease. Front. Neurosci..

[130] Valdinocci D., Kovarova J., Neuzil J., Pountney D.L. (2021). Alpha-Synuclein Aggregates Associated with Mitochondria in Tunnelling Nanotubes. Neurotox. Res..

[131] Mortimore G.E., Schworer C.M. (1977). Induction of autophagy by amino-acid deprivation in perfused rat liver. Nature.

[132] Mortimore G.E., Poso A.R. (1984). Lysosomal pathways in hepatic protein degradation: Regulatory role of amino acids. Fed. Proc..

[133] Nagy M., Fenton W.A., Li D., Furtak K., Horwich A.L. (2016). Extended survival of misfolded G85R SOD1-linked ALS mice by transgenic expression of chaperone Hsp110. Proc. Natl. Acad. Sci. USA.

[134] Roth D.M., Balch W.E. (2013). Q-bodies monitor the quinary state of the protein fold. Nat. Cell Biol..

[135] Liang X.H., Nichols J.G., Hsu C.W., Crooke S.T. (2021). Hsc70 Facilitates Mannose-6-Phosphate Receptor-Mediated Intracellular Trafficking and Enhances Endosomal Release of Phosphorothioate-Modified Antisense Oligonucleotides. Nucleic Acid Ther..

[136] Mollapour M., Bourboulia D., Beebe K., Woodford M.R., Polier S., Hoang A., Chelluri R., Li Y., Guo A., Lee M.J. (2014). Asymmetric Hsp90 N domain SUMOylation recruits Aha1 and ATP-competitive inhibitors. Mol. Cell.

[137] Wang B., Chen Z., Yu F., Chen Q., Tian Y., Ma S., Wang T., Liu X. (2016). Hsp90 regulates autophagy and plays a role in cancer therapy. Tumour. Biol..

[138] Schopf F.H., Biebl M.M., Buchner J. (2017). The HSP90 chaperone machinery. Nat. Rev. Mol. Cell Biol..

[139] Tanida I., Ueno T., Kominami E., Deretic V. (2008). LC3 and Autophagy. Autophagosome and Phagosome.

[140] Heras-Sandoval D., Perez-Rojas J.M., Hernandez-Damian J., Pedraza-Chaverri J. (2014). The role of PI3K/AKT/mTOR pathway in the modulation of autophagy and the clearance of protein aggregates in neurodegeneration. Cell Signal..

[141] Russell R.C., Tian Y., Yuan H., Park H.W., Chang Y.Y., Kim J., Kim H., Neufeld T.P., Dillin A., Guan K.L. (2013). ULK1 induces autophagy by phosphorylating Beclin-1 and activating VPS34 lipid kinase. Nat. Cell Biol..

[142] Jeon S.M. (2016). Regulation and function of AMPK in physiology and diseases. Exp. Mol. Med..

[143] Zachari M., Ganley I.G. (2017). The mammalian ULK1 complex and autophagy initiation. Essays Biochem..

[144] Khaminets A., Behl C., Dikic I. (2016). Ubiquitin-Dependent And Independent Signals In Selective Autophagy. Trends Cell Biol..

[145] Zheng Y.T., Shahnazari S., Brech A., Lamark T., Johansen T., Brumell J.H. (2009). The adaptor protein p62/SQSTM1 targets invading bacteria to the autophagy pathway. J. Immunol..

[146] Geisler S., Holmstrom K.M., Skujat D., Fiesel F.C., Rothfuss O.C., Kahle P.J., Springer W. (2010). PINK1/Parkin-mediated mitophagy is dependent on VDAC1 and p62/SQSTM1. Nat. Cell Biol..

[147] Mancias J.D., Wang X., Gygi S.P., Harper J.W., Kimmelman A.C. (2014). Quantitative proteomics identifies NCOA4 as the cargo receptor mediating ferritinophagy. Nature.

[148] Zhuang J., Ji X., Zhu Y., Liu W., Sun J., Jiao X., Xu X. (2021). Restriction of intracellular Salmonella typhimurium growth by the small-molecule autophagy inducer A77 1726 through the activation of the AMPK-ULK1 axis. Vet. Microbiol..

[149] Wang C., Wang H., Zhang D., Luo W., Liu R., Xu D., Diao L., Liao L., Liu Z. (2018). Phosphorylation of ULK1 affects autophagosome fusion and links chaperone-mediated autophagy to macroautophagy. Nat. Commun..

[150] Moreau K., Ravikumar B., Renna M., Puri C., Rubinsztein D.C. (2011). Autophagosome precursor maturation requires homotypic fusion. Cell.

[151] Kang R., Zeh H.J., Lotze M.T., Tang D. (2011). The Beclin 1 network regulates autophagy and apoptosis. Cell Death Differ..

[152] Lim J., Lachenmayer M.L., Wu S., Liu W., Kundu M., Wang R., Komatsu M., Oh Y.J., Zhao Y., Yue Z. (2015). Proteotoxic stress induces phosphorylation of p62/SQSTM1 by ULK1 to regulate selective autophagic clearance of protein aggregates. PLoS Genet..

[153] Menon M.B., Dhamija S. (2018). Beclin 1 Phosphorylation—at the Center of Autophagy Regulation. Front. Cell Dev. Biol..

[154] Popoff V., Mardones G.A., Bai S.K., Chambon V., Tenza D., Burgos P.V., Shi A., Benaroch P., Urbe S., Lamaze C. (2009). Analysis of articulation between clathrin and retromer in retrograde sorting on early endosomes. Traffic.

[155] Tabuchi M., Yanatori I., Kawai Y., Kishi F. (2010). Retromer-mediated direct sorting is required for proper endosomal recycling of the mammalian iron transporter DMT1. J. Cell Sci..

[156] Vilarino-Guell C., Wider C., Ross O.A., Dachsel J.C., Kachergus J.M., Lincoln S.J., Soto-Ortolaza A.I., Cobb S.A., Wilhoite G.J., Bacon J.A. (2011). VPS35 mutations in Parkinson disease. Am. J. Hum. Genet..

[157] Zimprich A., Benet-Pages A., Struhal W., Graf E., Eck S.H., Offman M.N., Haubenberger D., Spielberger S., Schulte E.C., Lichtner P. (2011). A mutation in VPS35, encoding a subunit of the retromer complex, causes late-onset Parkinson disease. Am. J. Hum. Genet..

[158] Deng H., Gao K., Jankovic J. (2013). The VPS35 gene and Parkinson’s disease. Mov. Disord..

[159] MacLeod D.A., Rhinn H., Kuwahara T., Zolin A., Di Paolo G., McCabe B.D., Marder K.S., Honig L.S., Clark L.N., Small S.A. (2013). RAB7L1 interacts with LRRK2 to modify intraneuronal protein sorting and Parkinson’s disease risk. Neuron.

[160] Jimenez-Orgaz A., Kvainickas A., Nagele H., Denner J., Eimer S., Dengjel J., Steinberg F. (2018). Control of RAB7 activity and localization through the retromer-TBC1D5 complex enables RAB7-dependent mitophagy. EMBO J..

[161] Zavodszky E., Seaman M.N., Moreau K., Jimenez-Sanchez M., Breusegem S.Y., Harbour M.E., Rubinsztein D.C. (2014). Mutation in VPS35 associated with Parkinson’s disease impairs WASH complex association and inhibits autophagy. Nat. Commun..

[162] Ishiguro M., Li Y., Yoshino H., Daida K., Ishiguro Y., Oyama G., Saiki S., Funayama M., Hattori N., Nishioka K. (2021). Clinical manifestations of Parkinson’s disease harboring VPS35 retromer complex component p.D620N with long-term follow-up. Parkinsonism Relat. Disord..

[163] Toffoli M., Vieira S.R.L., Schapira A.H.V. (2020). Genetic causes of PD: A pathway to disease modification. Neuropharmacology.

[164] King J.S., Gueho A., Hagedorn M., Gopaldass N., Leuba F., Soldati T., Insall R.H. (2013). WASH is required for lysosomal recycling and efficient autophagic and phagocytic digestion. Mol. Biol. Cell.

[165] Freeman C.L., Hesketh G., Seaman M.N. (2014). RME-8 coordinates the activity of the WASH complex with the function of the retromer SNX dimer to control endosomal tubulation. J. Cell Sci..

[166] Feng Y., Klionsky D.J. (2017). Autophagic membrane delivery through ATG9. Cell Res..

[167] Axe E.L., Walker S.A., Manifava M., Chandra P., Roderick H.L., Habermann A., Griffiths G., Ktistakis N.T. (2008). Autophagosome formation from membrane compartments enriched in phosphatidylinositol 3-phosphate and dynamically connected to the endoplasmic reticulum. J. Cell Biol..

[168] Lamb C.A., Joachim J., Tooze S.A., Galluzzi L., Bravo-San Pedro J.M., Kroemer G. (2017). Chapter Two—Quantifying Autophagic Structures in Mammalian Cells Using Confocal Microscopy. Methods in Enzymology.

[169] Kulkarni V.V., Maday S. (2018). Compartment-specific dynamics and functions of autophagy in neurons. Dev. Neurobiol..

[170] Patoli D., Mignotte F., Deckert V., Dusuel A., Dumont A., Rieu A., Jalil A., Van Dongen K., Bourgeois T., Gautier T. (2020). Inhibition of mitophagy drives macrophage activation and antibacterial defense during sepsis. J. Clin. Investig..

[171] Mallard F., Tang B.L., Galli T., Tenza D., Saint-Pol A., Yue X., Antony C., Hong W., Goud B., Johannes L. (2002). Early/recycling endosomes-to-TGN transport involves two SNARE complexes and a Rab6 isoform. J. Cell Biol..

[172] Winslow A.R., Chen C.W., Corrochano S., Acevedo-Arozena A., Gordon D.E., Peden A.A., Lichtenberg M., Menzies F.M., Ravikumar B., Imarisio S. (2010). alpha-Synuclein impairs macroautophagy: Implications for Parkinson’s disease. J. Cell Biol..

[173] Haack T.B., Hogarth P.H., Kruer M.C., Gregory A., Wieland T., Schwarzmayr T., Graf E., Sanford L., Meyer E., Kara E. (2012). Exome sequencing reveals de novo WDR45 mutations causing a phenotypically distinct, X-linked dominant form of NBIA. Am. J. Hum. Genet..

[174] Saitsu H., Nishimura T., Muramatsu K., Kodera H., Kumada S., Sugai K., Kasai-Yoshida E., Sawaura N., Nishida H., Hoshino A. (2013). De novo mutations in the autophagy gene WDR45 cause static encephalopathy of childhood with neurodegeneration in adulthood. Nat. Genet..

[175] Zhao Y.G., Sun L., Miao G., Ji C., Zhao H., Sun H., Miao L., Yoshii S.R., Mizushima N., Wang X. (2015). The autophagy gene Wdr45/Wipi4 regulates learning and memory function and axonal homeostasis. Autophagy.

[176] Cong Y., So V., Tijssen M.A.J., Verbeek D.S., Reggiori F., Mauthe M. (2021). WDR45, one gene associated with multiple neurodevelopmental disorders. Autophagy.

[177] Ji C., Zhao H., Li D., Sun H., Hao J., Chen R., Wang X., Zhang H., Zhao Y.G. (2020). Role of Wdr45b in maintaining neural autophagy and cognitive function. Autophagy.

[178] Wilson M.I., Dooley H.C., Tooze S.A. (2014). WIPI2b and Atg16L1: Setting the stage for autophagosome formation. Biochem. Soc. Trans..

[179] Gu Y., Princely Abudu Y., Kumar S., Bissa B., Choi S.W., Jia J., Lazarou M., Eskelinen E.L., Johansen T., Deretic V. (2019). Mammalian Atg8 proteins regulate lysosome and autolysosome biogenesis through SNAREs. EMBO J..

[180] Sora V., Kumar M., Maiani E., Lambrughi M., Tiberti M., Papaleo E. (2020). Structure and Dynamics in the ATG8 Family From Experimental to Computational Techniques. Front. Cell Dev. Biol..

[181] Maeda S., Otomo C., Otomo T. (2019). The autophagic membrane tether ATG2A transfers lipids between membranes. Elife.

[182] Dancourt J., Melia T.J. (2014). Lipidation of the autophagy proteins LC3 and GABARAP is a membrane-curvature dependent process. Autophagy.

[183] Tanji K., Mori F., Wakabayashi K. (2014). The Role of Atg8 Homologue in Lewy Body Disease. Autophagy: Cancer, Other Pathologies, Inflammation, Immunity, Infection, and Aging.

[184] Liu W.J., Ye L., Huang W.F., Guo L.J., Xu Z.G., Wu H.L., Yang C., Liu H.F. (2016). p62 links the autophagy pathway and the ubiqutin-proteasome system upon ubiquitinated protein degradation. Cell Mol. Biol. Lett..

[185] Su H., Wang X. (2011). Autophagy and p62 in cardiac protein quality control. Autophagy.

[186] Nihira K., Miki Y., Ono K., Suzuki T., Sasano H. (2014). An inhibition of p62/SQSTM1 caused autophagic cell death of several human carcinoma cells. Cancer Sci..

[187] Chang S., Kim J.H., Shin J. (2002). p62 forms a ternary complex with PKCzeta and PAR-4 and antagonizes PAR-4-induced PKCzeta inhibition. FEBS Lett..

[188] Jain A., Lamark T., Sjottem E., Larsen K.B., Awuh J.A., Overvatn A., McMahon M., Hayes J.D., Johansen T. (2010). p62/SQSTM1 is a target gene for transcription factor NRF2 and creates a positive feedback loop by inducing antioxidant response element-driven gene transcription. J. Biol. Chem..

[189] Birgisdottir A.B., Lamark T., Johansen T. (2013). The LIR motif—Crucial for selective autophagy. J. Cell Sci..

[190] Acharya M., Lingenfelter D.J., Huang L., Gage P.J., Walter M.A. (2009). Human PRKC apoptosis WT1 regulator is a novel PITX2-interacting protein that regulates PITX2 transcriptional activity in ocular cells. J. Biol. Chem..

[191] van der Zee J., Van Langenhove T., Kovacs G.G., Dillen L., Deschamps W., Engelborghs S., Matej R., Vandenbulcke M., Sieben A., Dermaut B. (2014). Rare mutations in SQSTM1 modify susceptibility to frontotemporal lobar degeneration. Acta Neuropathol..

[192] Niu Y., Zhang J., Dong M. (2021). Nrf2 as a potential target for Parkinson’s disease therapy. J. Mol. Med..

[193] Jiang P., Nishimura T., Sakamaki Y., Itakura E., Hatta T., Natsume T., Mizushima N. (2014). The HOPS complex mediates autophagosome-lysosome fusion through interaction with syntaxin 17. Mol. Biol Cell.

[194] Itakura E., Kishi-Itakura C., Mizushima N. (2012). The hairpin-type tail-anchored SNARE syntaxin 17 targets to autophagosomes for fusion with endosomes/lysosomes. Cell.

[195] Takats S., Pircs K., Nagy P., Varga A., Karpati M., Hegedus K., Kramer H., Kovacs A.L., Sass M., Juhasz G. (2014). Interaction of the HOPS complex with Syntaxin 17 mediates autophagosome clearance in Drosophila. Mol. Biol. Cell.

[196] Aoyagi K., Itakura M., Fukutomi T., Nishiwaki C., Nakamichi Y., Torii S., Makiyama T., Harada A., Ohara-Imaizumi M. (2018). VAMP7 Regulates Autophagosome Formation by Supporting Atg9a Functions in Pancreatic beta-Cells From Male Mice. Endocrinology.

[197] Tang B.L. (2019). Syntaxin 16’s Newly Deciphered Roles in Autophagy. Cells.

[198] Hatsuzawa K., Hirose H., Tani K., Yamamoto A., Scheller R.H., Tagaya M. (2000). Syntaxin 18, a SNAP receptor that functions in the endoplasmic reticulum, intermediate compartment, and cis-Golgi vesicle trafficking. J. Biol. Chem..

[199] Bennett M.K., Garcia-Arraras J.E., Elferink L.A., Peterson K., Fleming A.M., Hazuka C.D., Scheller R.H. (1993). The syntaxin family of vesicular transport receptors. Cell.

[200] Nair U., Jotwani A., Geng J., Gammoh N., Richerson D., Yen W.L., Griffith J., Nag S., Wang K., Moss T. (2011). SNARE proteins are required for macroautophagy. Cell.

[201] Moreau K., Renna M., Rubinsztein D.C. (2013). Connections between SNAREs and autophagy. Trends Biochem. Sci..

[202] Yoshida S., Hasegawa T., Suzuki M., Sugeno N., Kobayashi J., Ueyama M., Fukuda M., Ido-Fujibayashi A., Sekiguchi K., Ezura M. (2018). Parkinson’s disease-linked DNAJC13 mutation aggravates alpha-synuclein-induced neurotoxicity through perturbation of endosomal trafficking. Hum. Mol. Genet..

[203] Lee H.-J., Lee K.-H., Im H.-N. (2012). Interaction of Human α-Synuclein with VTI1B May Modulate Vesicle Trafficking. Bull. Korean Chem. Soc..

[204] Ishida Y., Nayak S., Mindell J.A., Grabe M. (2013). A model of lysosomal pH regulation. J. Gen. Physiol..

[205] Guzman J.N., Sanchez-Padilla J., Wokosin D., Kondapalli J., Ilijic E., Schumacker P.T., Surmeier D.J. (2010). Oxidant stress evoked by pacemaking in dopaminergic neurons is attenuated by DJ-1. Nature.

[206] Inoue I., Chiba M., Ito K., Okamatsu Y., Suga Y., Kitahara Y., Nakahara Y., Endo Y., Takahashi K., Tagami U. (2021). One-step construction of ferritin encapsulation drugs for cancer chemotherapy. Nanoscale.

[207] Xu Y.Y., Wan W.P., Zhao S., Ma Z.G. (2020). L-type calcium channels are involved in iron-induced neurotoxicity in primary cultured ventral mesencephalon neurons of rats. Neurosci. Bull..

[208] Verma A., Ravindranath V. (2019). CaV1.3 L-Type Calcium Channels Increase the Vulnerability of Substantia Nigra Dopaminergic Neurons in MPTP Mouse Model of Parkinson’s Disease. Front. Aging Neurosci..

[209] Yan H.F., Zou T., Tuo Q.Z., Xu S., Li H., Belaidi A.A., Lei P. (2021). Ferroptosis: Mechanisms and links with diseases. Signal. Transduct Target. Ther..

[210] Weiland A., Wang Y., Wu W., Lan X., Han X., Li Q., Wang J. (2019). Ferroptosis and Its Role in Diverse Brain Diseases. Mol. Neurobiol..

[211] Conrad M., Kagan V.E., Bayir H., Pagnussat G.C., Head B., Traber M.G., Stockwell B.R. (2018). Regulation of lipid peroxidation and ferroptosis in diverse species. Genes Dev..

[212] Goedert M. (2015). NEURODEGENERATION. Alzheimer’s and Parkinson’s diseases: The prion concept in relation to assembled Abeta, tau, and alpha-synuclein. Science.

[213] Valdinocci D., Radford R.A.W., Goulding M., Hayashi J., Chung R.S., Pountney D.L. (2018). Extracellular Interactions of Alpha-Synuclein in Multiple System Atrophy. Int. J. Mol. Sci..

[214] Jan A., Goncalves N.P., Vaegter C.B., Jensen P.H., Ferreira N. (2021). The Prion-Like Spreading of Alpha-Synuclein in Parkinson’s Disease: Update on Models and Hypotheses. Int. J. Mol. Sci..

[215] Ferreira N., Gonçalves N.P., Jan A., Jensen N.M., Van Der Laan A., Mohseni S., Vægter C.B., Jensen P.H. (2021). Trans-synaptic spreading of alpha-synuclein pathology through sensory afferents leads to sensory nerve degeneration and neuropathic pain. Acta Neuropathol. Commun..

[216] Van Den Berge N., Ferreira N., Mikkelsen T.W., Alstrup A.K.O., Tamguney G., Karlsson P., Terkelsen A.J., Nyengaard J.R., Jensen P.H., Borghammer P. (2021). Ageing promotes pathological alpha-synuclein propagation and autonomic dysfunction in wild-type rats. Brain.

[217] Ximerakis M., Pampalakis G., Roumeliotis T.I., Sykioti V.S., Garbis S.D., Stefanis L., Sotiropoulou G., Vekrellis K. (2014). Resistance of naturally secreted alpha-synuclein to proteolysis. FASEB J..

[218] Valdinocci D., Grant G.D., Dickson T.C., Pountney D.L. (2018). Epothilone D inhibits microglia-mediated spread of alpha-synuclein aggregates. Mol. Cell Neurosci..

[219] Xia Y., Zhang G., Han C., Ma K., Guo X., Wan F., Kou L., Yin S., Liu L., Huang J. (2019). Microglia as modulators of exosomal alpha-synuclein transmission. Cell Death Dis..

[220] Alvarez-Erviti L., Seow Y., Schapira A.H., Gardiner C., Sargent I.L., Wood M.J., Cooper J.M. (2011). Lysosomal dysfunction increases exosome-mediated alpha-synuclein release and transmission. Neurobiol. Dis..

[221] Salimi L., Akbari A., Jabbari N., Mojarad B., Vahhabi A., Szafert S., Kalashani S.A., Soraya H., Nawaz M., Rezaie J. (2020). Synergies in exosomes and autophagy pathways for cellular homeostasis and metastasis of tumor cells. Cell Biosci..

[222] Xu H., Wang Y., Song N., Wang J., Jiang H., Xie J. (2017). New Progress on the Role of Glia in Iron Metabolism and Iron-Induced Degeneration of Dopamine Neurons in Parkinson’s Disease. Front. Mol. Neurosci..

[223] Thomsen M.S., Andersen M.V., Christoffersen P.R., Jensen M.D., Lichota J., Moos T. (2015). Neurodegeneration with inflammation is accompanied by accumulation of iron and ferritin in microglia and neurons. Neurobiol. Dis..

[224] Jellinger K., Paulus W., Grundke-Iqbal I., Riederer P., Youdim M.B. (1990). Brain iron and ferritin in Parkinson’s and Alzheimer’s diseases. J. Neural Transm. Park. Dis. Dement. Sect..

[225] Shen X., Yang H., Zhang D., Jiang H. (2019). Iron Concentration Does Not Differ in Blood but Tends to Decrease in Cerebrospinal Fluid in Parkinson’s Disease. Front. Neurosci.

[226] Zhuang X.X., Wang S.F., Tan Y., Song J.X., Zhu Z., Wang Z.Y., Wu M.Y., Cai C.Z., Huang Z.J., Tan J.Q. (2020). Pharmacological enhancement of TFEB-mediated autophagy alleviated neuronal death in oxidative stress-induced Parkinson’s disease models. Cell Death Dis..

[227] Settembre C., Di Malta C., Polito V.A., Garcia Arencibia M., Vetrini F., Erdin S., Erdin S.U., Huynh T., Medina D., Colella P. (2011). TFEB links autophagy to lysosomal biogenesis. Science.

[228] Zhu Z., Yang C., Iyaswamy A., Krishnamoorthi S., Sreenivasmurthy S.G., Liu J., Wang Z., Tong B.C., Song J., Lu J. (2019). Balancing mTOR Signaling and Autophagy in the Treatment of Parkinson’s Disease. Int. J. Mol. Sci..

[229] Medina D.L., Di Paola S., Peluso I., Armani A., De Stefani D., Venditti R., Montefusco S., Scotto-Rosato A., Prezioso C., Forrester A. (2015). Lysosomal calcium signalling regulates autophagy through calcineurin and TFEB. Nat. Cell Biol..

[230] Surmeier D.J., Schumacker P.T. (2013). Calcium, bioenergetics, and neuronal vulnerability in Parkinson’s disease. J. Biol. Chem..

[231] Nath S., Goodwin J., Engelborghs Y., Pountney D.L. (2011). Raised calcium promotes alpha-synuclein aggregate formation. Mol. Cell Neurosci..

[232] Wera S., Neyts J. (1994). Calcineurin as a possible new target for treatment of Parkinson’s disease. Med. Hypotheses.

[233] Miller A.J., Levy C., Davis I.J., Razin E., Fisher D.E. (2005). Sumoylation of MITF and its related family members TFE3 and TFEB. J. Biol. Chem..

[234] Henley J.M., Craig T.J., Wilkinson K.A. (2014). Neuronal SUMOylation: Mechanisms, physiology, and roles in neuronal dysfunction. Physiol. Rev..

[235] Yan S., Sun X., Xiang B., Cang H., Kang X., Chen Y., Li H., Shi G., Yeh E.T., Wang B. (2010). Redox regulation of the stability of the SUMO protease SENP3 via interactions with CHIP and Hsp90. EMBO J..

[236] Fukuda I., Ito A., Hirai G., Nishimura S., Kawasaki H., Saitoh H., Kimura K., Sodeoka M., Yoshida M. (2009). Ginkgolic acid inhibits protein SUMOylation by blocking formation of the E1-SUMO intermediate. Chem. Biol..

[237] Vijayakumaran S., Nakamura Y., Henley J.M., Pountney D.L. (2019). Ginkgolic acid promotes autophagy-dependent clearance of intracellular alpha-synuclein aggregates. Mol. Cell Neurosci..

[238] Gerstmeier J., Seegers J., Witt F., Waltenberger B., Temml V., Rollinger J.M., Stuppner H., Koeberle A., Schuster D., Werz O. (2019). Ginkgolic Acid is a Multi-Target Inhibitor of Key Enzymes in Pro-Inflammatory Lipid Mediator Biosynthesis. Front. Pharmacol..

[239] Liu Y., Yang B., Zhang L., Cong X., Liu Z., Hu Y., Zhang J., Hu H. (2018). Ginkgolic acid induces interplay between apoptosis and autophagy regulated by ROS generation in colon cancer. Biochem. Biophys. Res. Commun..

[240] Baek S.H., Ko J.H., Lee J.H., Kim C., Lee H., Nam D., Lee J., Lee S.G., Yang W.M., Um J.Y. (2017). Ginkgolic Acid Inhibits Invasion and Migration and TGF-beta-Induced EMT of Lung Cancer Cells Through PI3K/Akt/mTOR Inactivation. J. Cell Physiol..

[241] Yao Q.Q., Li L., Xu M.C., Hu H.H., Zhou H., Yu L.S., Zeng S. (2018). The metabolism and hepatotoxicity of ginkgolic acid (17:1) in vitro. Chin. J. Nat. Med..

[242] Brackett C.M., Garcia-Casas A., Castillo-Lluva S., Blagg B.S.J. (2020). Synthesis and Evaluation of Ginkgolic Acid Derivatives as SUMOylation Inhibitors. ACS Med. Chem. Lett..

[243] Chaari A., Hoarau-Vechot J., Ladjimi M. (2013). Applying chaperones to protein-misfolding disorders: Molecular chaperones against alpha-synuclein in Parkinson’s disease. Int. J. Biol. Macromol..

[244] Riedel M., Goldbaum O., Schwarz L., Schmitt S., Richter-Landsberg C. (2010). 17-AAG induces cytoplasmic alpha-synuclein aggregate clearance by induction of autophagy. PLoS ONE.

[245] Vijayakumaran S., Wong M.B., Antony H., Pountney D.L. (2015). Direct and/or Indirect Roles for SUMO in Modulating Alpha-Synuclein Toxicity. Biomolecules.

[246] Cuervo A.M., Wong E. (2014). Chaperone-mediated autophagy: Roles in disease and aging. Cell Res..

